# Hybrid deep learning framework for real-time fault detection in squirrel-cage induction motors

**DOI:** 10.1371/journal.pone.0336323

**Published:** 2025-11-11

**Authors:** J. M. Jakaria, Jahin Sabir, Md. Zillur Rahman, Md. Feroz Ali

**Affiliations:** 1 Department of Electrical and Electronic Engineering, Faridpur Engineering College, Faculty of Engineering and Technology, University of Dhaka, Faridpur, Bangladesh; 2 Department of Electrical and Electronic Engineering, Pabna University of Science and Technology, Pabna, Bangladesh; Beijing Institute of Technology, CHINA

## Abstract

The Fourth Industrial Revolution has heightened the demand for intelligent and reliable predictive maintenance systems in industrial environments. This study proposes a hybrid deep learning-based framework for real-time fault detection in Squirrel-Cage Induction Motors (SCIMs). Utilizing eight deep learning architectures—CNN-GRU, CNN-LSTM, LSTM, BiLSTM, Stacked LSTM, GRU, CNN, and ANN—the framework was trained and tested on a comprehensive dataset comprising one million samples, evenly divided between healthy and faulty motor conditions. Hybrid models, particularly CNN-GRU and CNN-LSTM, achieved classification accuracies of 92.57% and 92.27%, respectively, outperforming the other baseline models across precision, recall, and F1-score by effectively capturing both temporal and spatial features. Beyond classification accuracy, the hybrids further demonstrated computational efficiency in terms of inference time, latency, and throughput, validating their suitability for real-time deployment. The system analyzes real-time sensor data, including torque, speed, power, and stator/rotor currents, to identify various fault types such as short circuits, overloads, mechanical failures, and open circuits. Developed in MATLAB Simulink, the framework demonstrates high accuracy and scalability for real-time deployment. While results are promising, the claims are positioned within the scope of the evaluated models, as direct benchmarking with state-of-the-art methods was not within the present scope. The framework demands substantial computing power and annotated datasets, yet it represents a step toward intelligent, self-aware industrial systems. Future work will focus on model optimization, deployment in resource-constrained environments, and validation with real-world noisy industrial data, explicitly considering sensor drift, varying load conditions, and fault severity levels across diverse motor types and operational scenarios. In addition, since bearing faults account for a significant share of induction motor failures in practice, it will be a key priority to ensure comprehensive industrial applicability.

## 1. Introduction

The rapid digital transformation of modern industries has introduced intelligent automation, data-driven decision-making, and real-time monitoring.[1,2]. Central to the smooth functioning of these automated systems are electric motors—especially SCIMs, which dominate industrial applications due to their simple design, low cost, durability, and ease of maintenance. These motors are extensively used in machinery such as pumps, compressors, fans, conveyors, and other critical equipment across sectors like manufacturing, mining, oil and gas, transportation, and water treatment [3]. However, the increasing operational demands and harsh working environments expose these motors to various mechanical and electrical faults over time.

Common faults in induction motors include stator winding short circuits, rotor bar breakages, bearing failures, phase imbalances, overload conditions, and open circuits [4,5]. These faults can occur due to excessive thermal stress, fluctuating load conditions, poor insulation, mechanical wear, or power quality issues. When undetected or left unaddressed, such faults may lead to severe consequences—including unplanned system downtime, increased maintenance costs, reduced operational efficiency, safety hazards, and in some cases, irreversible damage to machinery. In large-scale operations, even brief motor failures can result in significant financial and productivity losses.

To prevent such outcomes, timely and accurate fault detection is essential for ensuring the reliability, safety, and energy efficiency of industrial systems [6–8]. This has made condition monitoring and predictive maintenance indispensable in modern industrial practices. Traditional fault detection methods—such as vibration analysis, thermal imaging, current signature analysis, and model-based estimation techniques—have been widely employed for this purpose [9,10].While these methods are useful, they often require manual feature extraction, rely on expert interpretation, and may not perform well in detecting early-stage or multi-class faults under variable operating conditions.

With the rise of sensor-based automation and data availability, there is a growing interest in intelligent fault diagnosis systems capable of analyzing large volumes of real-time operational data. In particular, machine learning (ML) and deep learning (DL) approaches have emerged as promising tools for developing data-driven diagnostic systems that can automatically learn fault patterns and classify motor conditions with high accuracy [11,12]. However, the integration of such systems into real-world industrial settings poses several challenges, including handling noisy data, achieving real-time performance, ensuring scalability, and generalizing across diverse fault types and motor configurations [13–15]. This study is motivated by the pressing need for an intelligent, scalable, and robust diagnostic framework that can meet the evolving demands of Industry 4.0-driven environments.

Benninger et al. (2023) [16] in proposed a hybrid fault detection method for induction motors combining circuit modeling and ML, achieving 94.81% accuracy for non-bearing faults. While effective for electrical and mechanical issues, it failed to detect bearing faults. Future work suggests using vibration or acoustic data to address this limitation. Abdulkareem et al. (2025) [17] developed ML models for induction motor fault prediction, achieving accuracies of 91% Random Forest (RF), 90% Artificial Neural Network (ANN), K-Nearest Neighbors (k-NN), and 89% Decision Tree (DT). The study focused on limited fault types, potentially reducing generalizability. Future improvements include incorporating diverse fault conditions and datasets to enhance model robustness. Toma et al. (2020) [18] proposed a motor-current data-driven approach for induction motor bearing fault diagnosis, using statistical features and a genetic algorithm (GA) to select optimal features, achieving over 97% accuracy with KNN, DT, and RF classifiers. Limitations include focus on only inner and outer race faults, potentially limiting generalizability. Future work suggests incorporating frequency-domain analysis and diverse datasets to enhance robustness.

Wang et al. (2016) [19] proposed the IMPSO-RBFNN method, integrating an improved particle swarm optimization (PSO) with radial basis function neural network (RBFNN), achieving fault diagnosis accuracies of 99.00% (normal state), 98.64% (outer-race fault), 95.26% (inner-race fault), and 93.38% (rolling element fault) for motor bearings. Limitations include potential overfitting due to parameter optimization complexity. Future solutions involve exploring diverse datasets and advanced optimization techniques to enhance generalizability. Shirdel et al. (2023) [20] proposed a hybrid DL and ensemble method using sound signals for induction motor fault detection, achieving superior accuracy over state-of-the-art methods. Limited by sound signal sensitivity, future solutions include integrating multi-signal inputs and advanced denoising techniques for enhanced robustness. Hsueh et al. (2019) [21] proposed a Convolutional Neural Networks (CNN) based fault diagnosis system for induction motors using empirical wavelet transform, achieving higher accuracy than traditional methods for five fault types. Limited by reliance on current signals, future solutions include integrating multi-signal inputs and advanced feature extraction for enhanced robustness. Chang et al. (2019) [22] developed a condition monitoring system using a vibration-electrical hybrid approach, achieving superior fault classification accuracy for stator (56.21%), rotor, bearing, and misalignment faults compared to electrical-only detection (37.85% for stator). Limited by specific fault types, future solutions include multi-signal integration for broader applicability.

Martinez-Roman et al. (2021) [23] proposed an analytical model using a winding tensor approach for induction machines with multiple cage faults, achieving accurate fault diagnosis with reduced computational complexity. Limited to specific cage fault types, future solutions include integrating diverse fault scenarios and real-time data for enhanced applicability.

Khrakhuean et al. (2022) [24] developed a real-time induction motor health index prediction system using ML, with PSO achieving the highest accuracy (value not specified) among ANN, Gradient Boosted Trees (GBT), and RF models. Limited by specific petrochemical plant data, future solutions include diverse datasets and multi-fault scenarios for robustness. Terron-Santiago et al. (2021) [25] reviewed induction machine fault modeling techniques, highlighting Finite Element Method (FEM), analytical, and hybrid models for accurate fault simulation. No specific accuracy values were provided. Limited by computational complexity, future solutions include optimizing hybrid models and integrating real-time data for enhanced efficiency. Kumar et al. (2022) [26] reviewed conventional and AI-based fault diagnosis methods for induction motors, highlighting motor current signature analysis (MCSA) effectiveness. Bearing (44%) and winding (26%) faults dominate failures. Limitations include MCSA’s sensitivity to load variations; combining MCSA with AI techniques like deep learning offers improved accuracy and robustness. Barrera-Llanga et al. (2023) [27]compared six CNN architectures for detecting broken rotor bars in induction motors, achieving 99% accuracy with Visual Geometry Group (VGG)19, which excelled in precision, recall, and F1-score (0.994–0.998). Limitations include its focus on 28-bar motors and high computational demands. Future work suggests testing diverse motor designs and optimizing computational efficiency.

Bahgat et al. (2024) [28] reviewed fault detection techniques for three-phase induction motors, highlighting MCSA, partial discharge testing, and AI-based methods for detecting eccentricity (40% bearing defects), rotor, and stator faults. Limitations include complex operating conditions and noise interference. Solutions involve advanced AI, ML, and enhanced signal processing. Alqunun et al. (2025) [29] developed a hybrid ML framework for bearing fault detection, achieving 95.51% accuracy using ResNet-50-SVM with Continuous Wavelet Transform (CWT) and Tree-structured Parzen Estimator (TPE) for feature extraction and hyperparameter tuning. Compared to prior studies (e.g., Wen et al., 98.95%; Hou et al., 99.7%), it excels in generalization. Limitations include untested real-time applicability and potential inconsistencies across machines. Future work suggests evaluating computational burden and testing on diverse datasets. Kumar (2025) [30] reviewed transfer learning (TL) for induction motor health monitoring, emphasizing its effectiveness in addressing limited labeled data and diverse operating conditions. Studies like Wen et al. (98.95% accuracy) and Kumar et al. (99.43%) demonstrated TL’s superior fault detection. Limitations include domain mismatch and computational complexity. Solutions involve advanced domain adaptation and optimized TL frameworks. Chen et al. (2025) [31] proposed a BPNN model predicting electrode mass load, thickness, and porosity from AM content, viscosity, StoL, and CG. Limitations include NN black-box nature and limited quantitative electrode analysis; solutions involve PFI for feature importance and optimizing network depths. Mohammadi et al. (2025) [32] evaluated railway fastener defect detection using transfer learning, with ViT and DeiT achieving accuracy 95% and outperforming CNNs. Limitations include dataset constraints and high computational demands; solutions involve precise hyperparameter tuning and model selection for efficiency. Lu et al. (2022) [33] employed ViT for classifying ultrasonic B-scan images of rail defects, achieving accuracy over 90%. Limitations include reliance on basic ViT model due to time constraints; solutions involve dataset expansion and model fine-tuning for enhanced performance.

Past research on induction motor fault detection has explored a variety of methods, ranging from analytical modeling to ML and DL. Early hybrid approaches combining circuit modeling with ML achieved high accuracy for electrical and mechanical faults, though bearing faults often remained undetected. Several works used traditional algorithms such as RF, DT, GBT, and k-NN, producing solid results but relying heavily on manual feature extraction and focusing on a limited set of fault categories. Current-signal–based approaches, sometimes enhanced with genetic algorithms, delivered excellent performance for bearing faults but lacked coverage of diverse fault types. Optimization-driven neural networks and advanced feature engineering methods boosted performance in controlled settings, yet suffered from overfitting and reduced adaptability to varied industrial environments. DL models, particularly convolutional architectures, showed impressive fault classification accuracy, including in specialized cases such as broken rotor bar detection, but often at the cost of high computational requirements and limited generalization across motor designs. Hybrid sensing methods that combine vibration, electrical, and acoustic signals improved detection accuracy but faced challenges with sensor noise and integration complexity. Real-time frameworks have been proposed, but many are tested only on small, specific datasets, limiting their scalability. TL approaches have also emerged as a solution to data scarcity, achieving top-tier accuracy, yet issues such as domain mismatch and computational load remain. Beyond motor fault detection, recent studies have also applied deep learning to rail defect classification and lithium-ion battery electrode prediction, highlighting its cross-domain versatility and reinforcing the comprehensiveness of this review. Overall, while significant progress has been made, existing methods tend to focus on specific fault types, use modest datasets, and rarely combine spatial and temporal learning for large-scale, multi-fault, real-time applications.

The existing body of work predominantly addresses specific fault categories or single sensor modalities, rarely tackling the simultaneous detection of diverse electrical, mechanical, and supply related faults. In addition, the majority of prior studies have relied heavily on traditional ML techniques such as RF, DT, and k-NN which often require manual feature extraction and tend to struggle with complex, high dimensional datasets. Few approaches utilize large scale datasets, particularly those exceeding half a million samples, limiting statistical robustness and adaptability. Hybrid DL architectures capable of capturing both temporal and spatial dependencies are seldom employed, and detailed comparative evaluations across multiple model types are rare. Furthermore, considerations for real time deployment and computational efficiency remain insufficiently explored, highlighting the need for a more comprehensive and scalable solution.

Existing studies on induction motor fault diagnosis suffer from several methodological limitations. Most works concentrate on specific fault categories or rely on single-sensor modalities, failing to address simultaneous detection of diverse electrical, mechanical, and supply-related faults. The majority of approaches depend on traditional ML techniques such as RF, DT, and k-NN, which require manual feature extraction and struggle with high-dimensional, complex datasets. Although deep learning has been introduced, hybrid architectures that jointly capture temporal and spatial dependencies are rarely employed, and comparative evaluations across multiple model types are limited. Furthermore, many studies rely on small-scale datasets—often below half a million samples—restricting statistical robustness and generalizability. Real-time deployment aspects, scalability, and computational efficiency are also insufficiently explored, while reported high accuracies often lack validation in practical industrial contexts. Even advanced methods such as transfer learning or optimization-driven models remain computationally intensive and largely untested for deployment, underscoring a critical gap between controlled experimental research and industry-ready solutions.

**Key Contributions:**

- ***Multi-Fault Detection Capability*** – The framework is capable of detecting multiple categories of faults simultaneously, including electrical, mechanical, and supply-related faults, offering broader coverage than many prior studies that typically address only a single or narrow fault type.
- ***Hybrid Spatial–Temporal Modeling*** – By combining convolutional layers for spatial feature extraction with recurrent GRU/LSTM layers for temporal sequence learning, the framework captures richer fault signatures than standalone models, addressing a methodological gap in prior literature.
- ***Large-Scale Simulation-Driven Dataset*** – The system is trained and validated on a balanced dataset of one million samples generated through controlled simulations. While simulation-based, this scale of data collection ensures statistical robustness and enables realistic replication of diverse operating scenarios, a capability largely absent in earlier works.
- ***Comparative Model Benchmarking*** – A systematic evaluation across multiple deep learning models (ANN, CNN, LSTM, BiLSTM, Stacked LSTM, GRU, CNN–GRU, CNN–LSTM) identifies the most effective hybrid architecture, offering insights that are rarely provided in existing studies.
- ***Real-Time Industrial Alignment*** – Unlike many prior approaches limited to laboratory settings, this study embeds the framework within MATLAB Simulink with sensor-compatible configurations, bridging the gap between simulated research and real-world industrial deployment, and ensuring feasibility for monitoring and maintenance.

The purpose of this research is to design and implement an intelligent fault detection framework for SCIM that can operate reliably under a variety of industrial conditions. The research aims to develop a hybrid deep learning system capable of detecting and classifying multiple categories of faults covering electrical, mechanical, and supply related issues concurrently. By leveraging a large scale balanced dataset, the framework seeks to achieve high robustness and scalability, ensuring accurate performance across diverse operating scenarios. Another goal is to conduct a thorough comparative analysis of different deep learning models to identify the most effective architecture for spatial and temporal fault detection. Finally, the study focuses on ensuring real time operational capability with minimal hardware and computational requirements, thereby supporting predictive maintenance strategies that reduce downtime and improve overall industrial efficiency.

## 2. Methods & model

**Fig 1:**
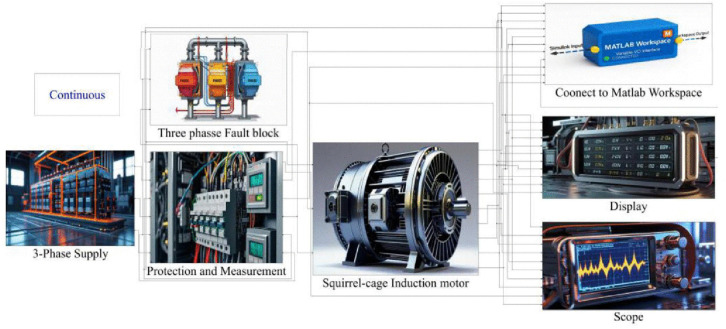
Three-Phase SCIM Simulink Schematic Diagram.

Fig 1 illustrates the simulation framework developed in MATLAB/Simulink for analyzing the performance and fault behavior of a SCIM. The setup begins with a three-phase supply feeding the motor system, followed by protection and measurement blocks to safeguard and monitor system parameters. A three-phase fault block is incorporated to introduce controlled fault scenarios, enabling the study of motor responses under abnormal operating conditions. The SCIM model represents the core machine, whose electrical and mechanical outputs are monitored through connected display and **s**cope blocks. Additionally, simulation data is linked to the MATLAB Workspace for advanced analysis and post-processing.

Table 1 presents the main specifications of the SCIM model used in the MATLAB/Simulink environment. The parameters—rated power, voltage, frequency, and speed—were selected to match a standard industrial motor, ensuring realistic behavior during simulation. This setup provides a reliable basis for supervised fault detection studies, enabling accurate performance evaluation and validation of monitoring algorithms under controlled conditions.

**Table 1:** Configuration of A SCIM.

Parameter	Value
Machine Type	Asynchronous Machine
Rotor Type	Squirrel-cage
Squirrel-Cage Preset Model	10 HP, 460 V, 60 Hz, 1760 RPM
Double Squirrel-Cage Model	Not used (left default/ open parameter estimator)

### 2.1. Fault simulation & analysis

To understand how an induction motor behaves when things go wrong, it is essential to study its performance under different faulty conditions [34]. In this work, we created a set of simulated scenarios that mimic common electrical and mechanical issues, allowing us to observe how each fault changes the motor’s electrical and mechanical characteristics. Using MATLAB/Simulink, we carefully introduced faults and monitored key parameters such as rotor current, stator current, torque, rotor speed, and output power over time. Starting with a healthy motor as a baseline, we then applied different types of faults, including phase-to-phase short circuits, phase-to-ground faults, overload conditions, mechanical disturbances, and open circuit faults. Each case was examined to identify unique patterns and changes in the waveforms, which later served as the foundation for developing a DL based fault detection system. By comparing faulty behavior to the healthy state, we could clearly see the signatures that each type of problem leaves behind, making it possible to detect them reliably in practice.

#### 2.1.1. Healthy condition.

**Fig 2:**
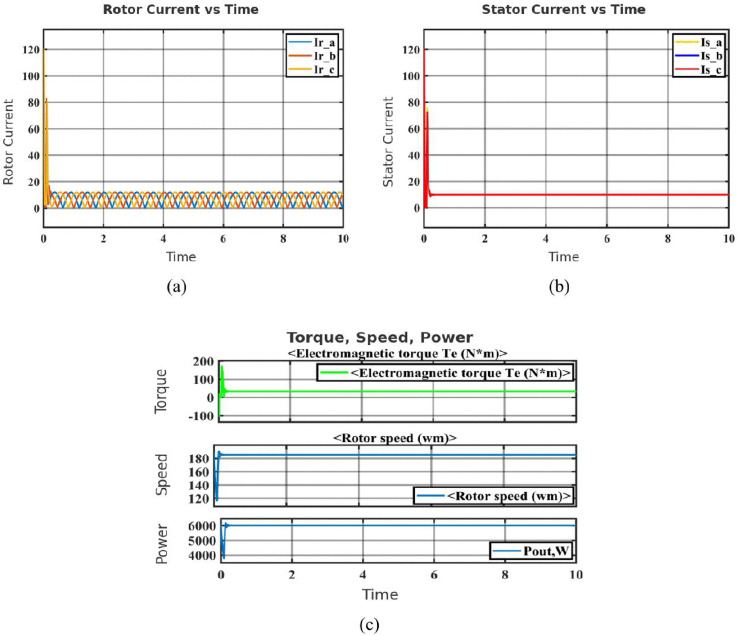
Healthy operating characteristics of the SCIM: (a) Rotor current in healthy motor (b) Stator current in healthy motor (c) Characteristics of healthy motor.

In the healthy motor condition, key parameters such as rotor current, stator current, electromagnetic torque, rotor speed and output power were recorded to establish a baseline. As shown in Fig 2(a), the rotor current experienced a brief inrush during startup before all phases stabilized into balanced sinusoidal waveforms. Similarly, Fig 2(b) shows the stator current with an initial magnetizing surge followed by a steady constant amplitude, confirming stable load conditions without electrical anomalies. Mechanical behavior in Fig 2(c) reveals a sharp torque rise at startup due to rotor inertia and friction, which subsided within 0.5 seconds as the rotor quickly reached synchronous speed. Output power briefly peaked during acceleration before stabilizing, indicating efficient energy conversion under steady operation.

#### 2.1.2. Short circuit fault.

Short circuit faults are critical electrical issues that can severely affect the performance and reliability of induction motors. These faults occur when unintended connections form between different phases or between a phase and the ground, causing abnormal current flows that disrupt the motor’s normal operation. Two common types of short circuit faults are phase-to-phase faults and phase-to-ground faults [35].

##### 2.1.2.1. Phase-to-phase fault:

**Fig 3:**
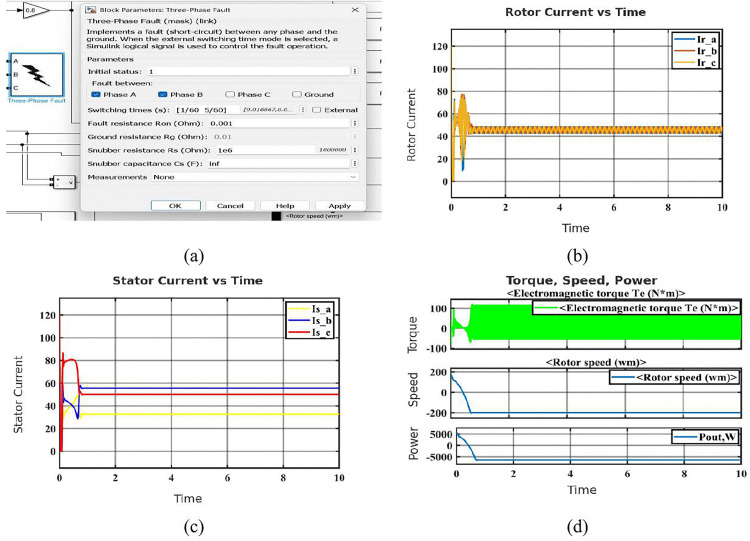
Simulation results of the SCIM under phase-to-phase fault condition: (a) Configuration block of phase-to-phase fault (phase A and phase B) (b) Rotor current in phase-to-phase fault condition (c) Stator current in phase-to-phase fault condition (d) Characteristics of phase-to-phase fault condition.

Phase-to-phase faults occur when two motor phases accidentally come into direct contact, creating a low-resistance path between them. This fault disrupts the normal current flow and magnetic balance in the motor, often causing severe current imbalances and mechanical disturbances [36]. The fault configuration block, seen in Fig 3(a), simulates a line-to-line fault between Phases A and B with extremely low resistance, which is perfect for testing worst-case situations. The rotor current waveforms (Ir_a, Ir_b, and Ir_c) in Fig 3(b) show imbalance and high-frequency transients just after the fault, suggesting a disruption in the rotor’s magnetic field and disturbed current flow. The waveforms of the stator current are shown in Fig 3(c). There is a noticeable imbalance following the fault, Phase C exhibits the maximum current because of the short circuit, whilst Phases A and B see a decrease in amplitude. This imbalance confirms aberrant stator activity and is indicative of inter-phase faults. The mechanical reaction of the system during a 10-second period, comprising output power (Pout), rotor speed (wm), and electromagnetic torque (Te), is shown in Fig 3(d). When a fault occurs, the torque exhibits a sudden overshoot followed by ongoing oscillations, which suggests that the torque stability has been lost. Reverse rotation is implied when the rotor speed steadily decreases and even goes negative. Concurrently, the output power drops, indicating that the motor has switched to regenerative braking and is resupplying power.

##### 2.1.2.2. Phase to ground fault:

**Fig 4:**
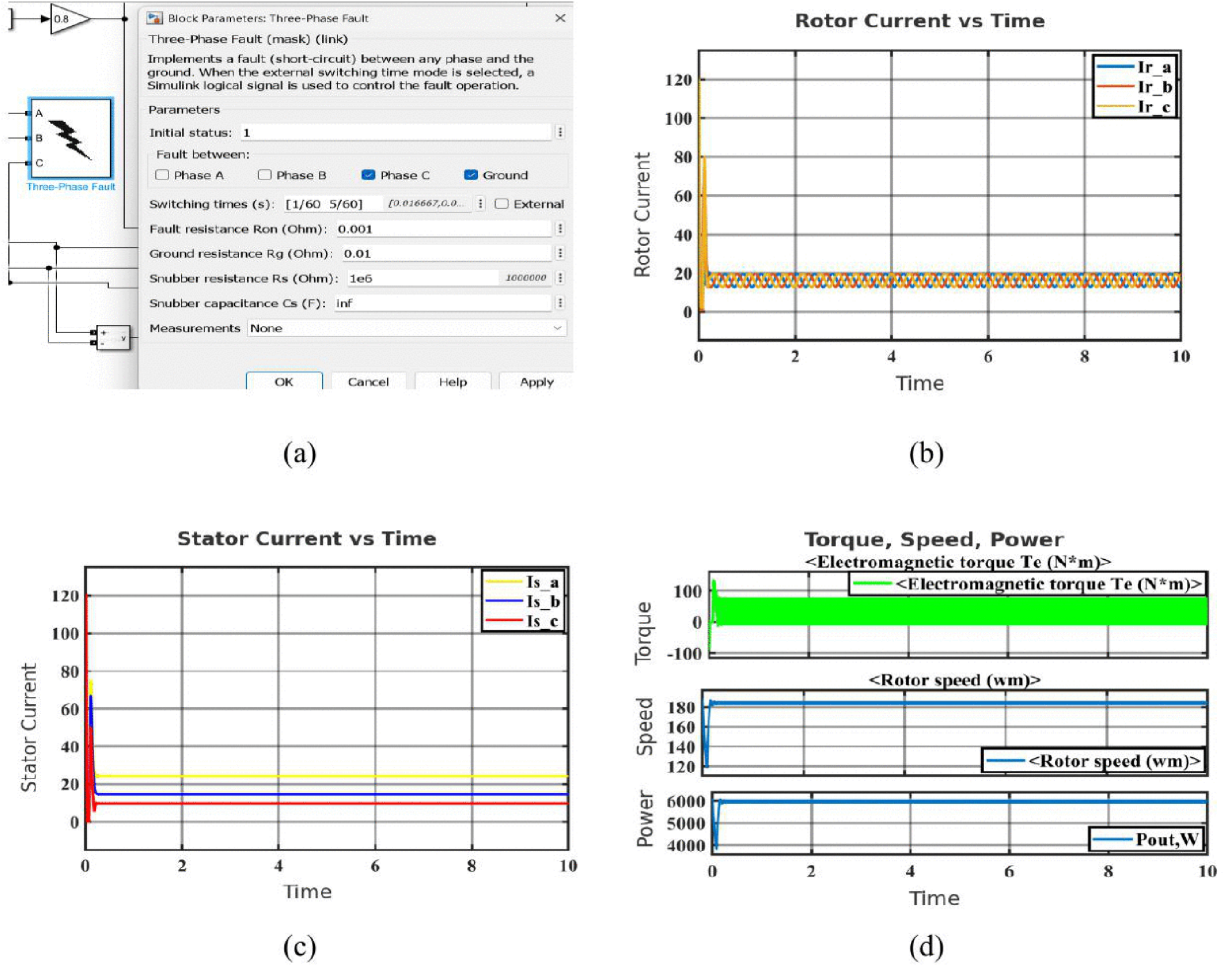
Simulation results of the SCIM under phase-to-ground fault condition: (a) Configuration block of phase to ground fault (phase C and Ground) (b) Rotor current in phase to ground fault condition (c) Stator current in phase to ground fault condition (d) Characteristics of phase to ground fault condition.

Phase-to-ground faults happen when a single phase unintentionally connects to the ground. This fault leads to leakage currents flowing to earth, resulting in distorted current waveforms and transient mechanical effects, but the motor may recover more quickly compared to phase-to-phase faults [37]. Fig 4(a) illustrates the setup that was utilized to apply the fault. There is a line-to-ground fault since only Phase C and Ground are chosen. Transient and steady-state responses are studied by simulating a severe fault thanks to the low resistance values. The Fig 4(b) displays particularly in Phase C, which was directly related to the problem, the rotor current exhibits a noticeable spike during the fault application. All three phase currents show minor distortion and sinusoidal behavior following the transient, demonstrating system recovery. The Fig 4(c) illustrates how the defect has a major impact on the stator current in Phase C. Because the fault route permits current to leak to ground, there is a significant initial spike followed by stability at a lower amplitude than during the healthy phases. The imbalance verifies the fault’s existence and location. Fig 4(d) shows the output power (Pout), rotor speed (wm), and electromagnetic torque (Te). The abrupt application of the fault causes a dramatic fluctuation in torque at the start of the simulation. The system does, however, swiftly settle, suggesting strong transient handling. The motor’s ability to recover synchronous speed after a malfunction is demonstrated by the first speed fluctuation. After a brief disruption, the power output likewise stabilizes, indicating that the fault’s long-term effects are modest.

#### 2.1.3. Overload fault.

**Fig 5:**
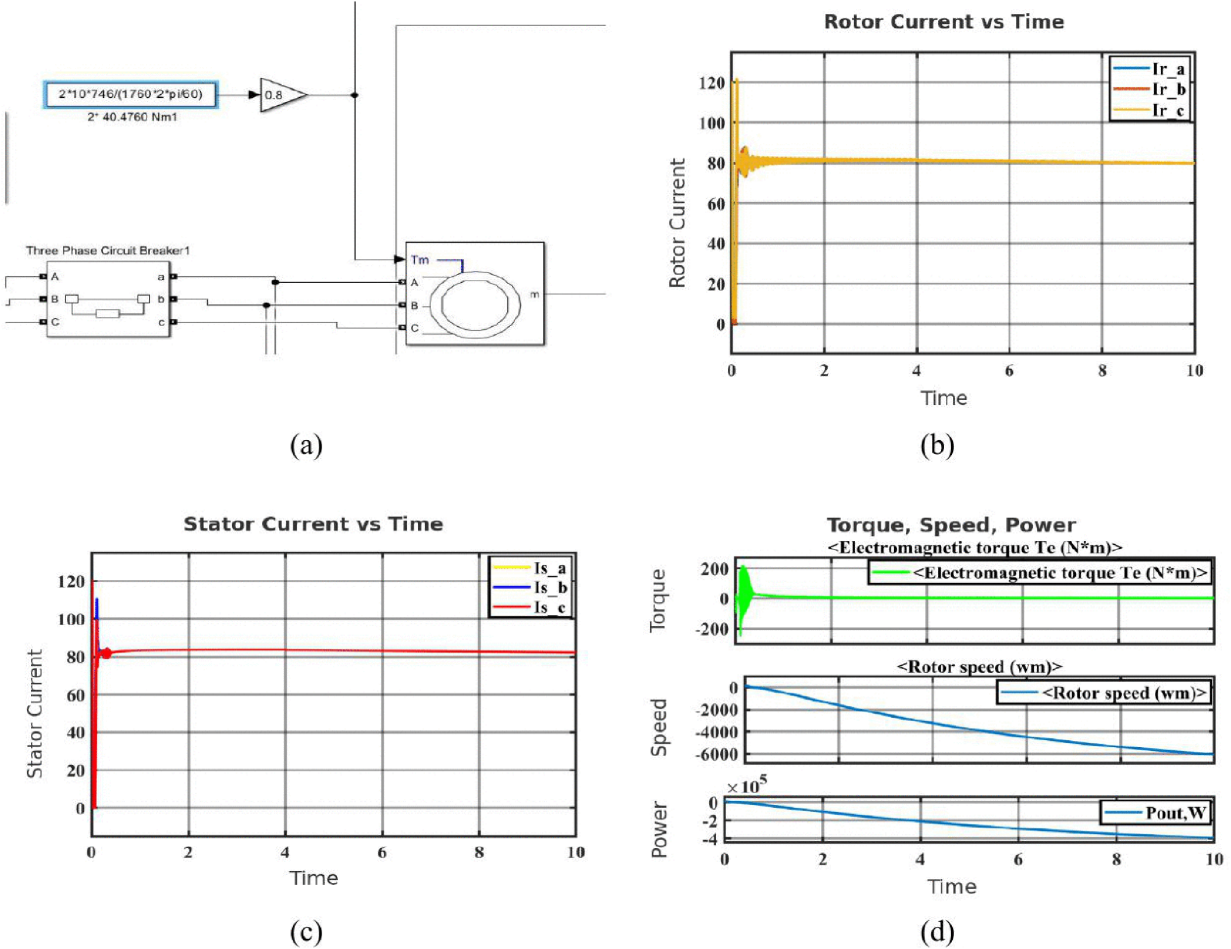
Simulation results of the SCIM under overload fault condition: (a) Overload fault Simulink (b) Rotor current in overload fault condition (c) Stator current in overload fault condition (d) Characteristics of overload fault condition.


Fig 5(a) illustrates the application of torque. Such a configuration enables the investigation of transitory behavior during fault clearing or sudden disconnection. To define the torque input, use the formula:

$$T=\frac{Pmech}{\frac{\text{2}\times \pi \times n_{s}}{\text{6}\text{0}}}$$ (1)

Where, T denotes the mechanical torque in Newton-meters(Nm), Pmech is the mechanical power, $n_{s}$ is synchronous speed, and π Mathematical constant pi (~3.1416). Here, during fault, T=2*T [38]. Fig 5(b) displays After a brief initial surge, the three rotor currents converge to a steady state value. Although there appears to be no fault between phases based on the symmetry of Ir_a, Ir_b, and Ir_c, the abrupt dynamic shift brought on by the disconnection is reflected in the huge spike at the onset. The Fig 5(c) displays The waveforms smooth down after the first half second, when the stator current likewise displays a very chaotic area. Is_a, Is_b, and Is_c gradually converge, suggesting that the motor circuit electrically stabilizes following the disconnect. The Fig 5(d) displays the power output, rotor speed, and electromagnetic torque following disconnect. After a brief period of high-frequency oscillations, the torque rapidly diminishes and settles around zero. This suggests that the motor has abruptly disconnected, meaning it is no longer producing mechanical output. When the rotor speed exhibits a negative exponential decline and reaches negative values, it means that system instability or regenerative dynamics is causing the rotor to slow down or reverse. The motor may have briefly become a generator owing to inertia before coming to a full stop if the power decreases sharply and goes negative.

#### 2.1.4. Mechanical fault.

**Fig 6:**
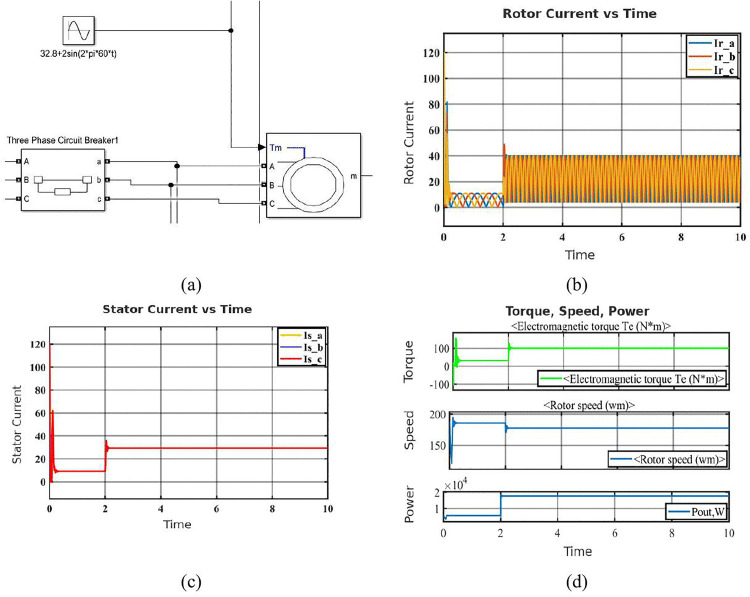
Simulation results of the SCIM under mechanical fault condition: (a) Mechanical fault Simulink (b) Rotor current in mechanical fault condition (c) Stator current in mechanical fault condition (d) Characteristics of mechanical fault condition.


The Fig 6(a) illustrates how a signal block in Simulink applies the torque signal:

$$T(t)=T+\text{2}\text{sin}(\text{2}\pi \times f_{s}\times t)$$ (2)

Which, T(t) represents the torque in Newton-meters (Nm) as a function of time t (in seconds), $f_{s}$ is the supply frequency (Hz). This configuration influences the motor’s electromagnetic and dynamic response by accurately simulating the varying torque demand seen during mechanical failures [39]. The rotor current (Ir_a, Ir_b, and Ir_c) waveforms in the Fig 6(b) exhibit a discernible rise in amplitude after the mechanical disturbance begins. The signals continue to be sinusoidal and balanced, suggesting that the disruption is mechanical in nature as opposed to electrical. The presence of a mechanical failure without any phase loss or imbalance is confirmed by the Fig 6(c), which demonstrates that the stator current (Is_a, Is_b, and Is_c) increases in amplitude across all phases while preserving waveform symmetry. The Fig 6(d) displays as the motor tries to counteract the varying mechanical stress, the electromagnetic torque significantly increases in magnitude. The system’s capacity to sustain operating speed in spite of the problem is demonstrated by the rotor speed’s relative stability. In proportion to the increased energy needed to overcome the mechanical disturbance, the motor’s output power increases.

#### 2.1.5. Open circuit fault.

**Fig 7:**
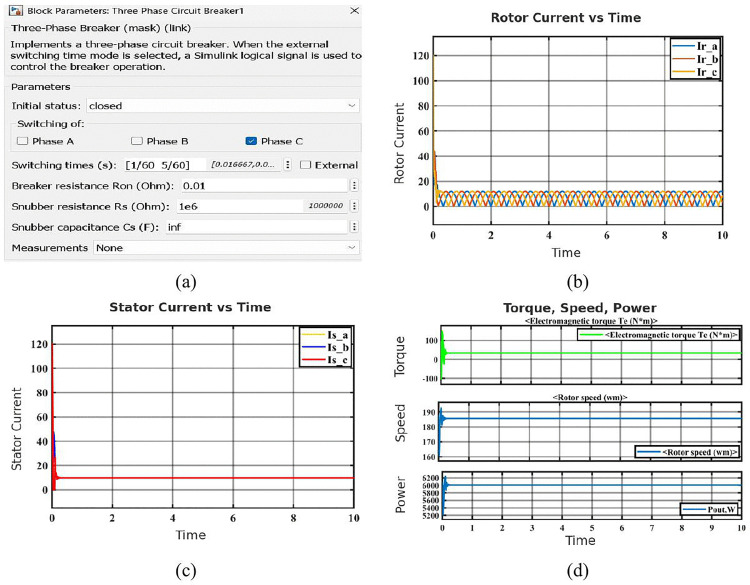
Simulation results of the SCIM under open-circuit fault condition: (a) Configuration block of open circuit fault (phase C) (b) Rotor current in open circuit fault condition (c) Stator current in open circuit fault condition (d) Characteristics of open circuit fault condition.

The Fig 7(a) illustrates how a three-phase breaker block in Simulink is used to apply an open circuit fault to Phase C of the induction motor. By briefly opening Phase C’s circuit while maintaining Phases A and B connected, the fault is introduced. The Fig 7(b) displays an imbalance can also be seen in the rotor currents. Ir_c drastically drops, but Ir_a and Ir_b continue to oscillate with only minor distortion. The missing phase current causes an uneven magnetic field, which is the cause of this. The stator current waveform in Fig 7(c) shows that as soon as the fault occurs, the current in Phase C (Is_c) goes to zero, indicating the open circuit. Phases A (Is_a) and B (Is_b) conduct in the meanwhile, but the system’s imbalance causes their waveforms to become distorted. At the beginning of the fault, the electromagnetic torque is disturbed, but it gradually stabilizes at a lower value in the Fig 7(d). While the output power is significantly decreased, the rotor speed stays nearly constant with just minimal variations, suggesting that the motor is still operating but performing at a lower level.

### 2.2. DL Model Implementation

**Fig 8:**
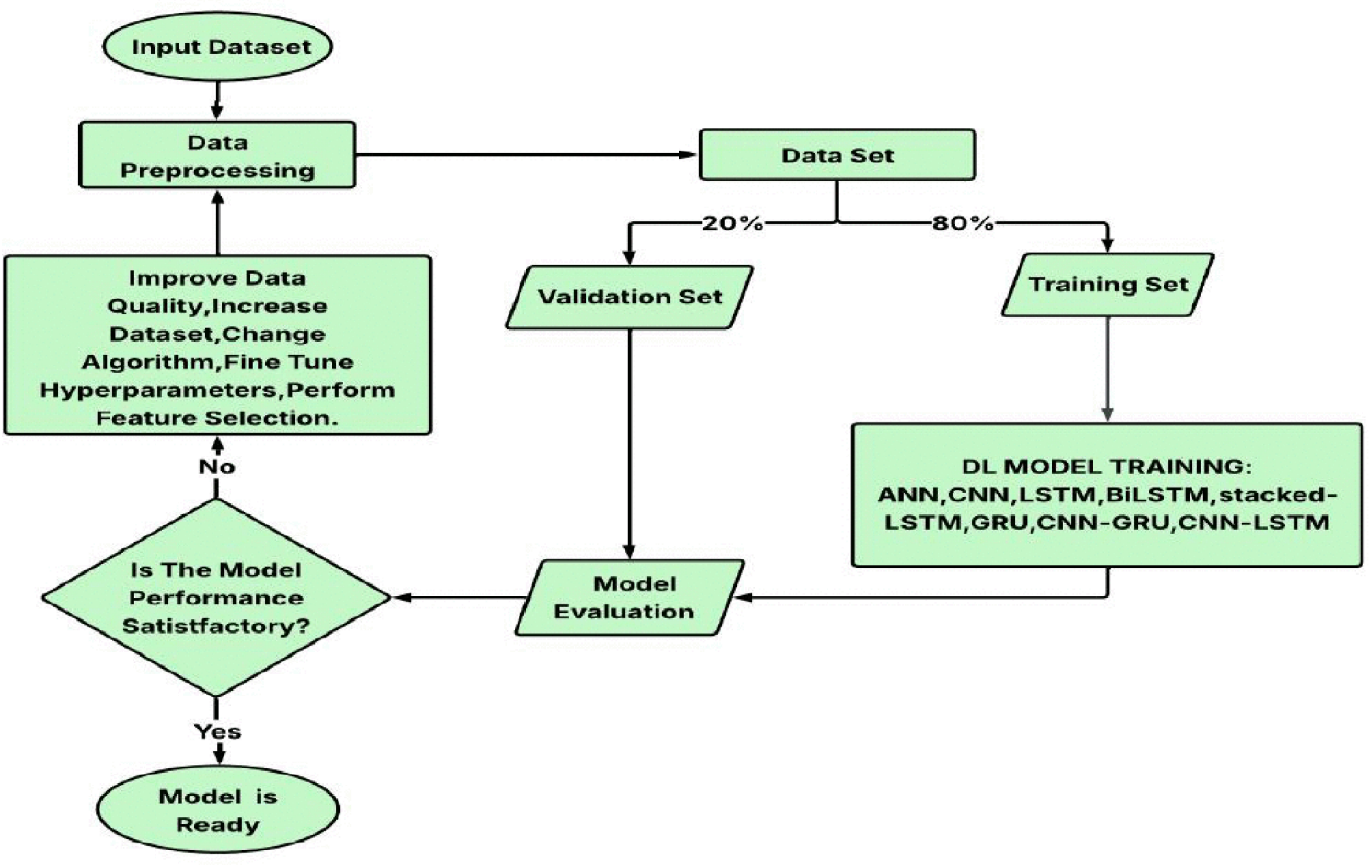
DL Process Flowchart.

The sequential procedure of creating and assessing a DL model for induction motor defect diagnostics is depicted in Fig 8. In order to enhance quality, expand the dataset, pick features, and adjust hyperparameters, the raw input dataset first goes through data preparation. Following processing, the dataset is divided into two parts: a 20% validation set and an 80% training set. The training set is used to train a variety of DL models, such as CNN, ANN, LSTM, BiLSTM (Bidirectional Long Short-Term Memory), stacked LSTM, GRU, CNN-GRU, and CNN-LSTM. Following training, the models’ performance is assessed using the validation set. The procedure goes back to enhancing data quality and adjusting model parameters if the model’s performance is subpar. The model is considered ready for deployment when this iterative cycle is completed and a suitable model performance is attained.

#### 2.2.1. Dataset generation.

Keeping a balanced 50–50% ratio between healthy and defective motor situations, our project’s goal was to gather a total of one million data points. In particular, within the first 10 seconds of the simulation, 500,000 data points were captured from a system running normally. With 100,000 data points assigned to each unique kind of failure, the remaining 500,000 data points were gathered under fault situations. Data was gathered under several load scenarios to guarantee the resilience of our model, including faults with different durations and intensities to increase variability and realism. By simulating a variety of operating circumstances, this method improved the model’s generalizability. During the data gathering process, we adhered to the following criteria:

- Currents in the Stator (Is_a, Is_b, and Is_c)-

$$I_{s\_a}=i_{s\_d}\text{cos}\theta_{s}-i_{s_{q}}\text{sin}\theta_{s}$$ (3)

$$I_{s\_b}=-\frac{\text{1}}{\text{2}}\left(i_{s\_d}\text{cos}\theta_{s}-i_{s\_q}\text{sin}\theta_{s}\right)+\frac{\sqrt{\text{3}}}{\text{2}}\left(i_{s\_d}\text{sin}\theta_{s}+i_{s\_q}\text{cos}\theta_{s}\right)$$ (4)

$$I_{s\_c}=-\frac{\text{1}}{\text{2}}\left(i_{s\_d}\text{cos}\theta_{s}-i_{s\_q}\text{sin}\theta_{s}\right)-\frac{\sqrt{\text{3}}}{\text{2}}\left(i_{s\_d}\text{sin}\theta_{s}+i_{s\_q}\text{cos}\theta_{s}\right)$$ (5)

Where, $I_{{s\_}_{a}},I_{s\_b},I_{s\_c}$ are stator phase currents, $i_{s\_d},i_{s\_q}$ are d-axis & q-axis stator currents, $\theta_{s}$ is the stator reference angle, and sin() is the trigonometric function.

- Currents in the Rotor (Ir_a, Ir_b, and Ir_c)-

$$I_{r\_a}=i_{r\_d}\text{cos}\theta_{r}-i_{r\_q}\text{sin}\theta_{r}$$ (6)

$$I_{r\_b}=-\frac{\text{1}}{\text{2}}\left(i_{r\_d}\text{cos}\theta_{r}-i_{r\_q}\text{sin}\theta_{r}\right)+\frac{\sqrt{\text{3}}}{\text{2}}\left(i_{r\_d}\text{sin}\theta_{r}+i_{r\_q}\text{cos}\theta_{r}\right)$$ (7)

$$I_{r\_c}=-\frac{\text{1}}{\text{2}}\left(i_{r\_d}\text{cos}\theta_{r}-i_{r\_q}\text{sin}\theta_{r}\right)-\frac{\sqrt{\text{3}}}{\text{2}}\left(i_{r\_d}\text{sin}\theta_{r}+i_{r\_q}\text{cos}\theta_{r}\right)$$ (8)

Where, $I_{{r\_}_{a}\;},\;\;I_{r\_b}\;,{\;I}_{r\_c}$ are rotor phase currents, $i_{r\_d}\;,\;i_{r\_q}$ are d-axis & q-axis rotor currents, .$\theta_{r}$ is the rotor reference angle, and cos() is the trigonometric function.

- Input Power-

$$P_{\text{in}}=\sqrt{\text{3}}\cdot V_{\text{L}}\cdot I_{\text{line}}\cdot \text{cos}\;\varphi$$ (9)

Where, $P_{\text{in}}$ is the input power, $V_{\text{L}}$ is the line to line voltage, $I_{\text{line}}$ is line current, and cosφ is the power factor.

- Rotor Frequency-

$$f_{r}=s\cdot f_{s}$$ (10)

Where, $f_{r}$ is the rotor frequency, s is the slip, and $f_{s}$ is the supply frequency (Hz).

- Rotor Torque-

$$T_{r}=\frac{Pmech}{\frac{\text{2}\times \pi \times n_{s}}{\text{6}\text{0}}}$$ (11)

Where, $T_{r}$ denotes the rotor torque in Newton-meters(Nm), Pmech is the mechanical power, $n_{s}$ is synchronous speed, and π Mathematical constant pi (~3.1416).

- Rotor Speed-

$$N_{r}=(\text{1}-s)\cdot n_{s}$$ (12)

Where, $N_{r}$ is the rotor speed, s is the slip, and $n_{s}$ is synchronous speed.

- Efficiency-

$$\eta =\frac{P_{\text{out}}}{P_{\text{in}}}\times \text{1}\text{0}\text{0}\%$$ (13)

Where, η is the efficiency, $P_{\text{out}}$ is output power, $P_{\text{in}}$ is input power.

- Level (1, 2, 3, 4, 5, 6)- Six different motor conditions are represented by the levels 1, 2, 3, 4, 5 and 6 in this study: Level 1 represents a healthy condition; Level 2 refers to a phase-to-phase fault; Level 3 indicates a phase-to-ground fault; Level 4 represents an overload fault; Level 5 refers to a mechanical fault; and Level 6 indicates an open circuit fault.

**Fig 9:**
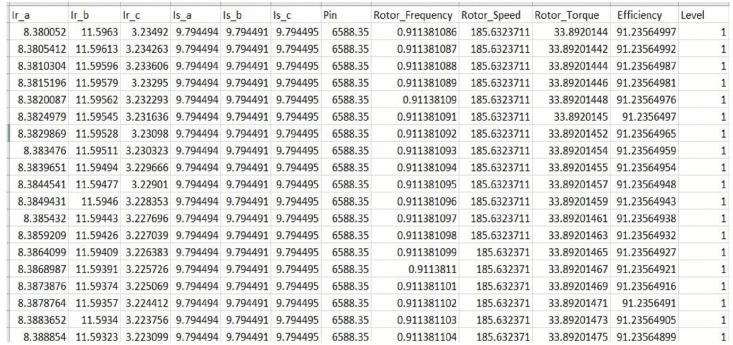
Partial Generated Dataset.

The dataset consists of 1,000,000 samples and 12 features, with no missing values. In Fig 9, an example of the created dataset is displayed.

**Fig 10:**
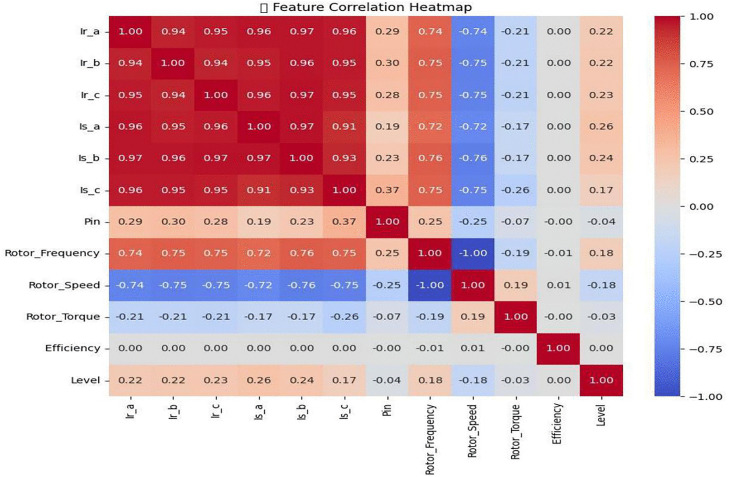
Feature Correlation Heat-map.

As seen in Fig 10, a Feature correlation heatmap was created to assess the interdependency between characteristics and the target fault classes. Redundancy is indicated by the extremely high positive correlation (r > 0.94) between the current signals (Ir_a, Ir_b, Ir_c, Is_a, Is_b, and Is_c). Rotor speed and Rotor frequency both have a strong negative correlation (r ≈ −1.00), suggesting that they effectively transmit the opposite information. Features like Efficiency and Level show little to no association, indicating that they might not be directly helpful for categorization. In order to improve model performance, this analysis aids in guiding feature selection and dimensionality reduction.

#### 2.2.2. Data preprocessing.

An essential component of every DL research is data preprocessing. It all comes down to ensuring that the data we use is high-quality and appropriate for creating prediction models [40]. To increase the dataset’s dependability, we used a number of preprocessing methods to the raw data gathered from the SCIMs for our investigation.

- ***Resolving the Data Issues*:** We started by addressing any missing or erratic sensor readings. Ignoring these problems might lead to bias and impair model performance. When it made sense, we filled in the blanks with imputation. To maintain the dataset’s cleanliness and dependability, we eliminated entries where the data couldn’t be accurately assessed.
- ***Selecting the Best Features:*** We selected the most pertinent facts from the vibration data to aid the model in comprehending motor failures. These comprised variance, RMS (Root Mean Square), mean, standard deviation, and other fundamental statistical characteristics. To extract information from the frequency domain, we also employed the Fourier Transform. We have a robust collection of signals to train the model on, thanks to this blend.
- ***Making the Data Uniform:*** The value ranges of several attributes might differ greatly. Bigger numbers might unfairly affect the model if we don’t change that. We employed Min-Max normalization to resolve the issue, ensuring that every feature falls inside the same range. This improved the accuracy of the model’s learning.

#### 2.2.3. Data splitting.

We divide the data into two parts: eighty percent is used for training the model and the remaining twenty percent is kept aside for testing. This means the model learns from most of the data available. The reason for doing this is to give the model enough examples so that it can understand different situations it might face in real life. When the model trains on a large portion of the data, it can recognize patterns more effectively. During the training phase, the model only sees the eighty percent of data assigned for training and has no access to the remaining twenty percent. Once training is complete, we use the test data to evaluate how well the model performs on new and unseen information. Testing with separate data helps us identify any errors the model might make and ensures that it will work well not just on the data it has seen before but also on new data it encounters later.

#### 2.2.4. Model training.

We used a variety of DL models that could recognize both temporal and spatial patterns in sensor data in order to tackle the difficulty of failure identification in induction motors. CNNs were utilized to extract local characteristics [41], while ANNs were employed as a baseline [42]. Time dependencies were addressed by the LSTM and BiLSTM models, whereas Stacked LSTM provided more in-depth temporal learning [43]. A lightweight substitute with similar sequence modeling capabilities was offered by GRU. Furthermore, for improved performance, hybrid models such as CNN-GRU and CNN-LSTM integrated sequential and spatial learning [44–46]. These architectures worked together to create a strong foundation for precise and effective predictive maintenance. An outline of the key characteristics of each model is provided here.

##### 2.2.4.1. ANN:

ANNs consist of layers of connected neurons. Each neuron performs a simple calculation by multiplying inputs by weights, adding a bias, and applying an activation function to introduce non-linearity. This process is repeated across the layers, allowing the network to learn complex patterns from the data. Mathematically, this is represented as:


$$y=f\left(\sum {\mathrm{\backslash nolimits}}_{i=\text{1}}^{n}\;w_{i}x_{i}+b\right)$$ (14)

Where, $x_{i}$ are the input values, $w_{i}$ are the weights, b is the bias, f is the activation function, and y is the output value. In this model, the input layer takes 11 scaled features, followed by a hidden layer with 128 neurons using the ReLU (Rectified Linear Unit) activation function to capture non-linear relationships. To prevent overfitting, a dropout layer disables 30% of neurons randomly during training. The second hidden layer contains 64 ReLU neurons, helping with efficient learning. The output layer uses the softmax activation function to classify multiple fault categories. The Adam optimizer, known for its flexible learning rate, was used to train the model with a batch size of 128 over 10 epochs. The model’s performance was evaluated using accuracy and loss metrics [47,48].

##### 2.2.4.2. CNN:

CNNs are effective at identifying local patterns and spatial features from structured inputs like spectrograms or modified vibration data. CNNs use convolutional layers where filters slide over the input data to create feature maps. Mathematically, the value at position (i,j) in the feature map is calculated by summing the element-wise multiplication of a filter K with a corresponding section of the input X.


$$F(i,j)=\sum {\mathrm{\backslash nolimits}}_{m}\;\sum {\mathrm{\backslash nolimits}}_{n}\;X(i+m,j+n)\cdot K(m,n)$$ (15)

Where, X(i+m,j+n) represents the input values in the local region, and K(m,n) represents the filter weights. In this model, the CNN starts with a one-dimensional convolutional layer that has 64 filters, each with a size of 3, and uses the ReLU activation function to capture local patterns among the 11 input features. This is followed by a dense layer with 64 neurons and a flatten layer to prepare the data for classification. The output layer applies the softmax function for multi-class classification. The model was trained using the Adam optimizer with categorical cross-entropy loss for 10 epochs and a batch size of 128 [49–51].

##### 2.2.4.3. LSTM:

A particular kind of RNN (Recurrent Neural Networks) called an LSTM network is made to simulate sequential dependencies, it is perfect for temporal vibration data. Using input, forget, and output gates, each LSTM unit keeps its cell state $C_{t}$ updated:


$$C_{t}=f_{t}*C_{t-\text{1}}+i_{t}*\text{tanh}\left(W_{C}\cdot \left[h_{t-\text{1}},x_{t}\right]+b_{C}\right)$$ (16)

$$h_{t}=o_{t}*\text{tanh}\left(C_{t}\right)$$ (17)

Where, $C_{t}$ is the cell state at time t, $C_{t-\text{1}}$ is the previous cell state, $W_{C}$ is the weight matrix for the candidate cell state, $b_{C}$ is the bias for the candidate cell state, tanh is the hyperbolic tangent activation function, $h_{t}$ is the hidden state at time, and $o_{t}$ is the output gate vector. The temporal dependencies in the sequential input data were intended to be captured by this LSTM model. To learn time-based patterns across the 11-feature input, it had a single 64-unit LSTM layer. ReLU activated a dense layer of 64 neurons to improve feature transformation. Multi-class categorization was made possible by the final softmax output layer. In order to ensure effective sequence learning and generalization, the model was trained across 10 epochs with a batch size of 128 using the Adam optimizer with categorical_crossentropy loss [52].

##### 2.2.4.4. BiLSTM:

By processing the input sequence both forward and backward, BiLSTM goes beyond the conventional LSTM. This greatly improves its capacity to identify patterns in motor activity that could rely on events rather than isolated points by taking into account both past and future context at each time step.


$$\vec{h_{t}}=\text{LSTM}\left(x_{t},\vec{h_{t-\text{1}}}\right)$$ (18)

$$\overset{\leftarrow }{h_{t}}=\text{LSTM}\left(x_{t},\overset{\leftarrow }{h_{t+\text{1}}}\right)$$ (19)

$$h_{t}=\left[\vec{h_{t}};\overset{\leftarrow }{h_{t}}\right]$$ (20)

Where, $\vec{h_{t}}$ is the hidden state at time t moving forward in the sequence, $x_{t}$ is the input at time t, $\vec{h_{t-\text{1}}}$ is the previous hidden state in the forward direction, $\overset{\leftarrow }{h_{t}}$ is the hidden state at time t moving backward in the sequence, $\overset{\leftarrow }{h_{t+\text{1}}}$ is the next hidden state in the backward direction, and $h_{t}$ is the combined hidden state at time t. This dual viewpoint facilitates a more sophisticated interpretation of vibration signals for fault identification, which improves the categorization of subtle or overlapping fault patterns [53–55].

##### 2.2.4.5. Stacked LSTM:

A deep LSTM architecture known as “Stacked LSTM” has several LSTM layers stacked on top of each other. While later layers pick up more intricate and abstract patterns, the first layer extracts low-level temporal information from the input data.


$$h_{t}^{\left(\text{1}\right)}={\text{LSTM}}^{\left(\text{1}\right)}\left(x_{t}\right)$$ (21)

$$h_{t}^{\left(\text{2}\right)}={\text{LSTM}}^{\left(\text{2}\right)}\left(h_{t}^{\left(\text{1}\right)}\right)$$ (22)

Where, $x_{t}$ is the input at time t, $h_{t}^{\left(\text{1}\right)}$ is the hidden state output from the first layer, and $h_{t}^{\left(\text{2}\right)}$ is the hidden state from the second layer. This layered architecture enhances the model’s ability to identify both early and advanced failure signals, making it especially helpful for modeling long-range relationships in noisy or high-frequency motor signal data [56,57].

##### 2.2.4.6 GRU:

GRUs simplify the LSTM structure by combining some of its gates, making the model lighter and faster to train. Despite this simplification, GRUs still effectively capture temporal relationships in sequential data.


$$h_{t}=\left(\text{1}-z_{t}\right)*h_{t-\text{1}}+z_{t}*{\tilde{h}}_{t}$$ (23)

Where, $z_{t}$ is the update gate, ${\tilde{h}}_{t}$ is the candidate hidden state created using the reset gate and current input, $h_{t-\text{1}}$ is the hidden state from the previous time step, and $h_{t}$ is the final hidden state. GRUs provide an excellent balance between computational economy and performance for induction motor fault detection, which makes them perfect for situations requiring rapid fault diagnosis without compromising accuracy [58].

##### 2.2.4.7. CNN-GRU:

The CNN-GRU model is a hybrid DL approach that combines the strengths of GRUs for modeling time sequences and CNNs for extracting spatial features. This combination is particularly effective for diagnosing faults in induction motors, as the signal data often contains both time-dependent patterns, such as regular fluctuations caused by bearing wear or mechanical issues, and localized features like sudden spikes in amplitude or specific frequency harmonics.


$$\text{Output}\;\text{state},\;\;h_{t}=\left(\text{1}-z_{t}\right)⊙h_{t-\text{1}}+z_{t}⊙{\tilde{h}}_{t}$$ (24)

Where, $h_{t}$ is the updated hidden state at time t, $z_{t}$ is the update gate, $h_{t-\text{1}}$ is the hidden state from the previous time step, ${\tilde{h}}_{t}$ is the candidate hidden state, and ⊙ denotes element-wise multiplication. The model is able to identify issues that manifest as localized temporal disturbances in the motor signal profile because of this design [59].

##### 2.2.4.8. CNN-LSTM:

LSTM and CNN are combined in the CNN-LSTM model to capture long-term temporal dependencies and spatial correlations in induction motor data. CNN layers are employed up front to identify significant patterns like high-frequency spikes, recurrent oscillations, or anomalies that point to eccentricity defects, overload, or mechanical misalignment. LSTM layers, which are more expressive than GRU and have a larger range for retaining past fault progression, get the CNN’s output after that.


$$\text{Final}\;\text{output}\;\text{states},\;h_{t}=o_{t}⊙\text{tanh}\left(c_{t}\right)$$ (25)

Where, $h_{t}$ is the updated hidden state at time t, $c_{t}$ is the current cell state, $o_{t}$ is the output gate, ⊙ denotes element-wise multiplication, and tanh is the hyperbolic tangent activation function. CNN-LSTM is appropriate for complicated induction motor failure situations because of its deep temporal representation, which increases its sensitivity in detecting both abrupt and gradually building defects [60,61].

#### 2.2.5. Model hyper-parameters.

The preprocessed sequences were grouped into batches of 128 samples and iteratively trained over 10 epochs with learning rate 0.001. Table 2. shows various neural network model architectures, where the structure of each layer is utilized. The models include ANN, CNN, LSTM, BiLSTM, Stacked LSTM, GRU, CNN-GRU, and CNN-LSTM. Each model architecture displays the layers (such as Dense, Conv1D, Dropout, Flatten, etc.) along with their dimensions or parameters, determined in relation to the specified batch size, epochs, and learning rate.

**Table 2:** Layer architectures of DL models.

Models	Layer architectures
ANN	Dense(128) → Dropout(0.3) → Dense(64) → Dense(6)
CNN	Conv1D(64,kernel = 3) → Flatten → Dense(64) → Dense(6)
LSTM	LSTM(64) → Dense(64) → Dense(6)
BiLSTM	Bidirectional(LSTM(64)) → Dense(64) → Dense(6)
Stacked LSTM	LSTM(64, return_seq = True) → LSTM(32) → Dense(64) → Dense(6)
GRU	GRU(64) → Dense(64) → Dense(6)
CNN-GRU	Conv1D(64,kernel = 3) → GRU(64) → Dense(6)
CNN-LSTM	Conv1D(64,kernel = 3) → LSTM(64) → Dense(6)

#### 2.2.6. Model inspection.

We assessed the models using a range of performance indicators, such as accuracy, precision, recall, and the F1-score, after the training process. These measurements provide a thorough evaluation of the models’ ability to forecast motor behavior and identify issues.

- ***Accuracy:*** To assess the performance of our models, we employed the accuracy metric, as shown by equation 26. In relation to all instances, it measures the percentage of accurately predicted cases in the testing dataset, taking into account both true positives (TP) and true negatives (TN). A higher accuracy score indicates that the model’s predictions are more accurate [17,62].

$$\text{Accuracy}=\frac{\text{TP}+\text{TN}}{\left(TP+TN+FP+FN\right)}$$ (26)

- ***Precision:*** Another important measure is precision, which is shown by equation 27. Out of all the positive predictions the model produced, it determines the proportion of accurate predictions. In situations when false positives (FP) might have serious repercussions, precision is especially important [17,62].

$$\text{Precision}=\frac{TP}{TP+FP}$$ (27)

- ***Recall:*** Also referred to as true positive rate or sensitivity, recall assesses how effectively our models accurately forecast positive occurrences [17,62]. It calculates the ratio of genuine positive predictions to all actual positive occurrences in the testing dataset, as shown in equation 28.

$$\text{Recall}=\frac{TP}{TP+FN}$$ (28)

- ***F1-Score:*** The F1-score provides a comprehensive evaluation of the predictive maintenance models by combining precision and recall. By calculating their harmonic average, it strikes a compromise between recall and accuracy. Because it offers a comprehensive assessment of the model’s efficacy, this statistic is especially helpful when both positive and negative classes are significant [17,62]. Equation 29 illustrates how the F1-score is determined.

$$\text{F1}-\text{Score}=\text{2}\times \frac{Precision\times Recall}{Precision+Recall}$$ (29)

By classifying predictions into true positives (TP), true negatives (TN), false positives (FP), and false negatives (FN), the confusion matrix offers a comprehensive analysis of our models’ performance and is another crucial performance indicator that we used. By contrasting the genuine levels with the predicted labels for every classification job, the confusion matrix was created to assess the models’ performance. For every motor state, the matrix breaks out the right and wrong categories.

To evaluate the suitability of the proposed framework for real-time deployment, we assessed additional computational efficiency metrics. Specifically, we measured latency, inference time, throughput, and memory usage. These metrics provide insight into the framework’s computational efficiency, responsiveness, and resource requirements, which are critical for deploying the model practically in real-time.

- ***Latency:*** Latency is the total time a model takes to produce an output after receiving an input. It includes all delays, such as data preprocessing, model computation, and post-processing. Low latency is important for real-time applications to ensure quick responses [63].
- ***Inference Time:*** Inference time measures only the time taken by the model to perform forward computation, excluding other overheads like preprocessing or post-processing. It reflects the pure computational efficiency of the model [64].
- ***Throughput:*** Throughput indicates how many samples a model can process per seconds, often measured in inferences or frames per second. Higher throughput means the model can handle larger amounts of data efficiently, which is important for industrial or large-scale use [65].

$$\text{Throughput}=\frac{n}{t}$$ (30)

Where, n is the total number of samples and t is the process time (sec).

- ***Memory Usage:*** Memory usage refers to the total computational memory a model requires, including memory for model parameters, intermediate activations, and input data. Optimizing memory usage is crucial for deployment on resource-limited devices like embedded systems or edge devices [66,67].

#### 2.2.7. Computational considerations.

All models were trained and tested on an Asus ROG Zephyrus G15 GA503RM system with an Intel Core i9-12900H CPU (14 cores), NVIDIA GeForce RTX 3080 Laptop GPU (8 GB GDDR6 VRAM), and 32 GB DDR5 RAM, running Linux-6.6.97 with Python 3.12.11 and TensorFlow 2.19.0. Training each deep learning architecture (ANN, CNN, LSTM, BiLSTM, Stacked LSTM, GRU, CNN-GRU, CNN-LSTM) on one million samples required approximately 2–2.5 hours, depending on complexity. Benchmarking functions integrated in the code reported GPU memory usage of 5–7 GB and system RAM consumption of 18–22 GB. Inference metrics showed per-batch latency in the range of 101–111 ms across all models, with throughput values between 1148 and 1255 samples/s. These results establish a clear hardware threshold and computational cost profile, thereby addressing deployment feasibility for industrial applications.

## 3. Results & discussion

This section explores the findings of our in-depth investigation as well as the knowledge acquired by using ML approaches to forecast the behavior and malfunctions of induction motors. Our research efforts have resulted in a multitude of insightful findings that provide insight into how well these models work in industrial settings. In order to give a thorough evaluation of the predictive maintenance models, we will go over the data in depth, going over accuracy, precision, recall, F1-score, and the confusion matrix.

One million samples in all, taken from induction motors running in a range of situations, make up the dataset utilized in this investigation. Each condition is represented by an equal number of samples, with 500,000 samples allocated to motors in good condition and 500,000 samples allocated to motors in bad condition, further divided among several fault kinds. 80% of the dataset was used for training, while 20% was used for testing, in order to guarantee reliable model training and assessment. This amounts to 800,000 training samples in total, split across the conditions, with 400,000 samples going to motors in good condition and 400,000 to motors in bad condition. Similar to this, there are 200,000 samples in the testing set, with 100,000 samples for each motor state (healthy and faulty).

### 3.1. Model evaluation

#### 3.1.1. ANN model.

**Fig 11:**
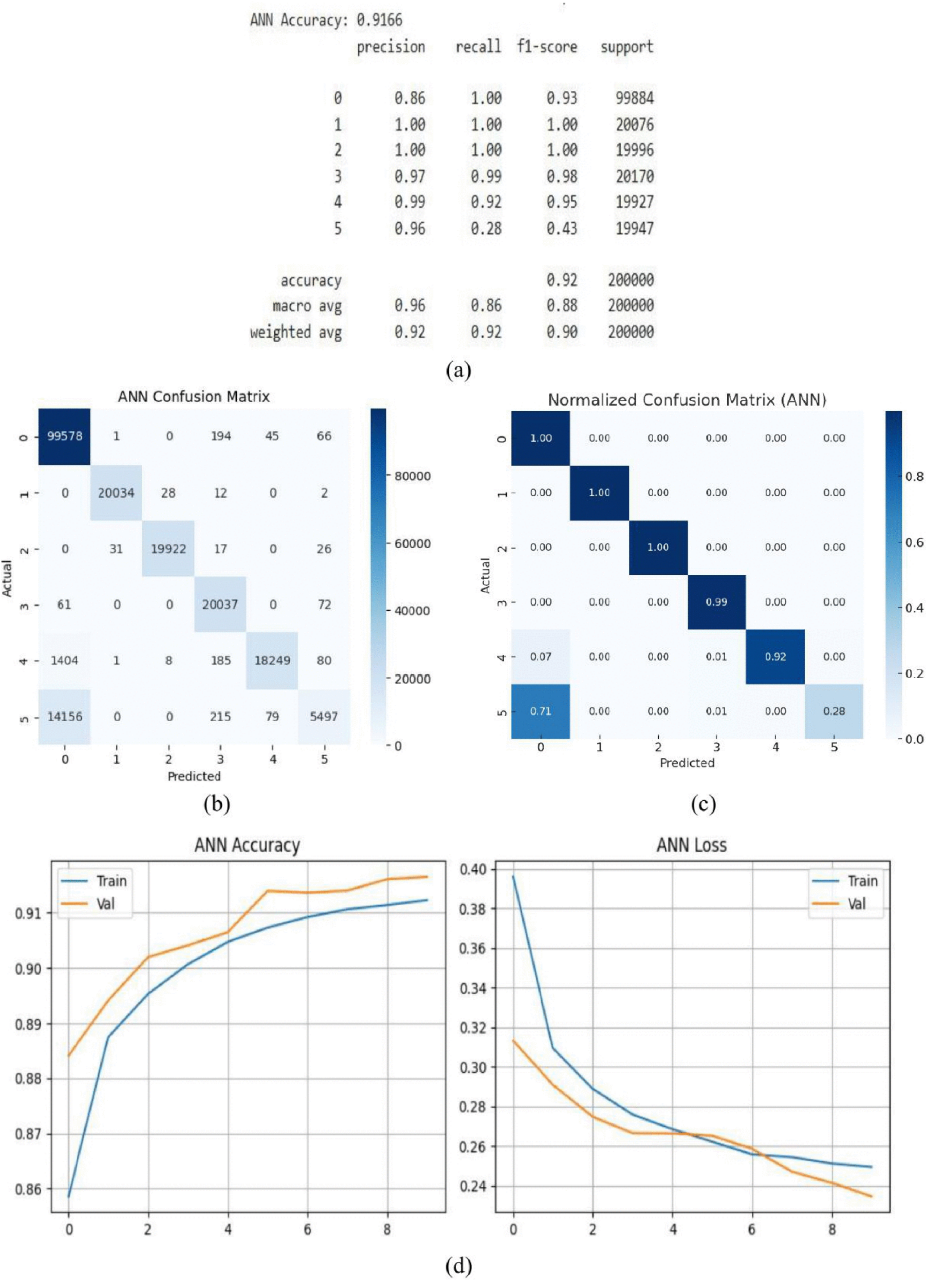
ANN model (a) summary (b) confusion matrix (c) normalized confusion matrix (d)accuracy & loss curve.

With an accuracy of 91.66% after 10 training epochs, the ANN model demonstrated impressive performance, accurately classifying the great majority of input data. The model’s ability to learn and recognize the intricate patterns in the dataset is demonstrated by this high degree of accuracy. Metrics such as accuracy, recall, and F1-score, which varied from 0.86 to 1.0 across all classes, were used to further validate the model’s performance, as shown in Fig 11(a). These strong results attest to the model’s capacity to produce forecasts that are both balanced and incredibly accurate across a range of fault scenarios. A more thorough analysis of the model’s predictions in the confusion matrix, Fig 11(b) provides insightful information about how well it performed. The ANN model is positioned as a potent tool for complex pattern recognition and accurate prediction in real-world applications due to its capacity to attain such precision and its constant near-perfect matches across cases. Fig 11(c) shows the normalized confusion matrix of the ANN model. Unlike the raw confusion matrix, normalization represents the proportion of correct and incorrect predictions for each class, making classification performance easier to interpret. The diagonal values correspond to the recall of each class, while the off-diagonal values indicate misclassification rates. For example, Class 0, 1, and 2 achieve perfect recall (1.00), Class 4 reaches 0.92, and Class 5 achieves 0.28, with most of its misclassified samples assigned to Class 0. Overall accuracy computed from the matrix which consistent with the reported performance metrics. The ANN model’s training and validation accuracy as well as loss throughout ten epochs are depicted in Fig 11 (d). Good generalization is shown by the accuracy graph’s consistent increase, where validation accuracy marginally surpasses training accuracy. Additionally, the loss curve shows a steady decreasing trend, indicating appropriate convergence without overfitting.

#### 3.1.2. CNN Model.

**Fig 12:**
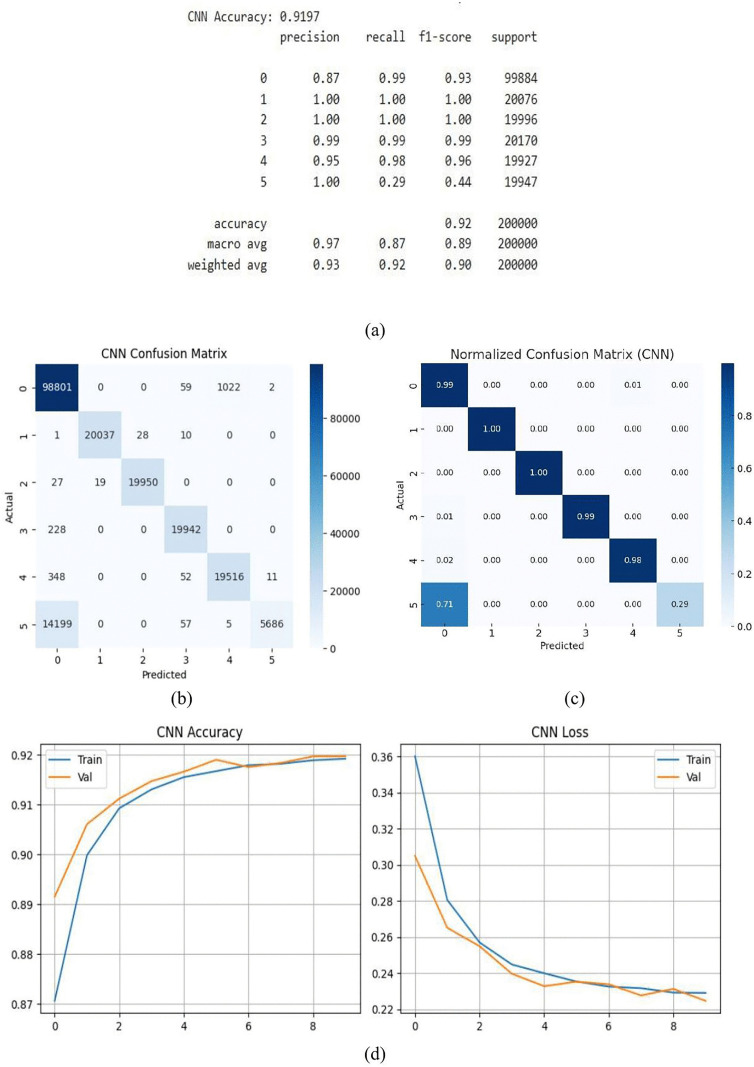
CNN model (a) summary (b) confusion matrix (c) normalized confusion matrix (d) accuracy & loss curve.

With a 91.97% accuracy rate, the CNN model successfully recognized patterns and correctly classified the majority of examples. With accuracy, recall, and F1-scores ranging from 0.87 to 1.0, as seen in Fig 12(a), it demonstrated consistent and dependable performance across all fault types. The model’s predictions are further illuminated by the confusion matrix in Fig 12(b), which shows that the model’s classification is almost flawless in many situations. CNN’s proficiency in identifying intricate patterns is further supported by this. The CNN model’s normalized confusion matrix is displayed in Fig 12(c). Normalization makes classification results easier to understand by showing the percentage of accurate and inaccurate predictions for each class, in contrast to the raw confusion matrix. Each class’s recall is represented by the diagonal numbers, while misclassification rates are shown by the off-diagonal values. A recall of 0.99 is attained by Class 0, 1.00 by Class 1 and 2, 0.99 by Class 3, and 0.98 by Class 4. Class 5 on the other hand has a poor recall of 0.29, with the majority of its incorrectly categorized samples (0.71) being assumed to be Class 0. The matrix’s overall accuracy was calculated in accordance with the performance parameters that were supplied. The validation and training curves are displayed in Fig 12(d). With validation loss marginally less than training loss, the accuracy plot progressively increases to 91.97% while the loss curve continuously decreases, indicating steady training and no overfitting.

#### 3.1.3. LSTM model.

**Fig 13:**
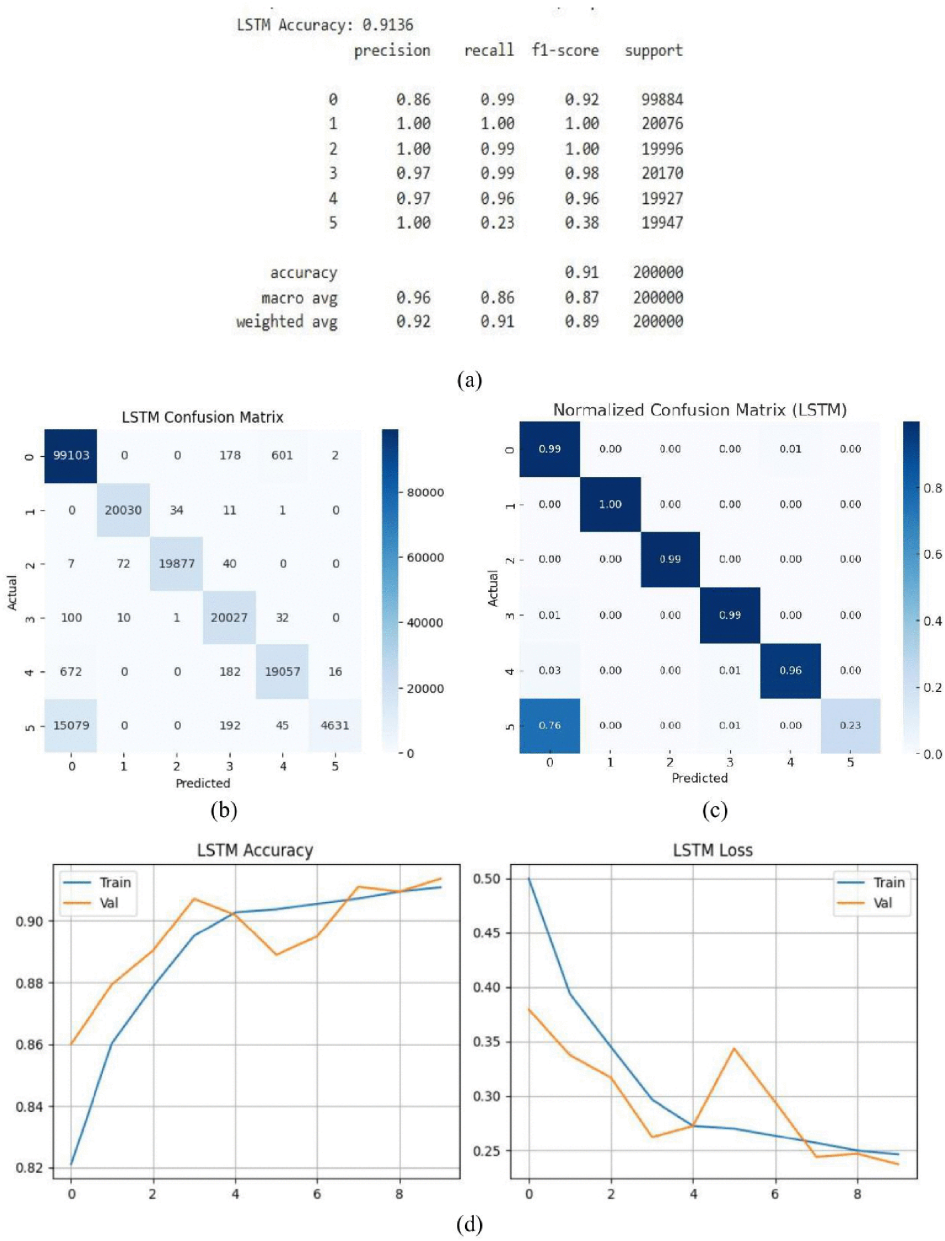
LSTM model (a) summary (b) confusion matrix (c) normalized confusion matrix (d) accuracy & loss curve.

LSTM model produced remarkable outcomes with a remarkable 91.36% accuracy rate. The model’s excellent capacity to accurately categorize the great majority of data after training is shown in its high performance. This precision demonstrates how well LSTM can identify and understand the intricate patterns concealed in the dataset. This degree of accuracy demonstrates how well the model captures complex data relationships and produces incredibly trustworthy predictions, particularly when used with the original information. The precision, recall, and F1-scores for each class range from 0.86 to 1.0, as illustrated in Fig 13(a), provide an overview of the model’s post-training performance. This highlights the model’s reliable performance in a range of scenarios. Fig 13(b). displays with its detailed breakdown of the prediction results and improved visualization of the model’s performance, the confusion matrix provides additional insight. All things considered, the LSTM model shows itself to be a potent instrument for complicated pattern recognition and accurate prediction in practical applications because to its high accuracy and constant near-perfect scores across cases. The LSTM model’s normalized confusion matrix is displayed in Fig 13(c). Normalization makes classification results easier to understand by showing the percentage of accurate and inaccurate predictions for each class, in contrast to the raw confusion matrix. Each class’s recall is represented by the diagonal numbers, while misclassification rates are shown by the off-diagonal values. For example, Class 0 achieves a recall of 0.99, Class 1 reaches 1.00, Class 2 records 0.99, Class 3 attains 0.99, and Class 4 achieves 0.96. In contrast, Class 5 shows a low recall of 0.23, with most of its misclassified samples (0.76) predicted as Class 0. Overall accuracy computed from the matrix consistent with the reported performance metrics. The LSTM model’s accuracy and loss performance are illustrated in Fig 13(d). Strong learning capacity is indicated by the close relationship between the training and validation accuracy, which peaks at 91.36%. A steady decline in the loss values indicates that the model has picked up useful temporal information from the data.

#### 3.1.4. BiLSTM model.

**Fig 14:**
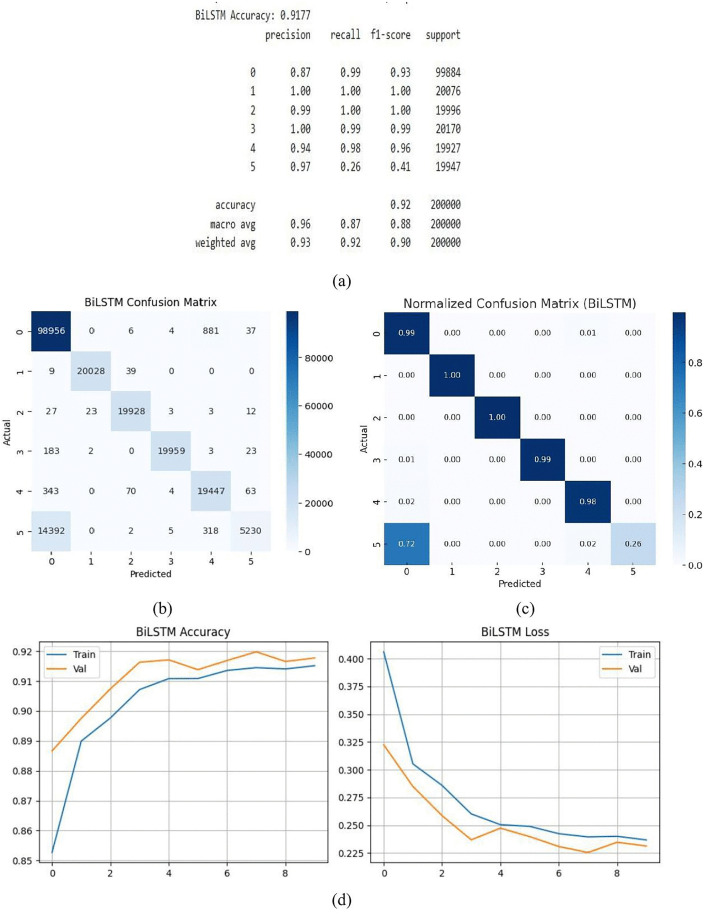
BiLSTM model (a) summary (b) confusion matrix (c) normalized confusion matrix (d)accuracy & loss curve.

The BiLSTM model successfully classified the majority of cases with a high accuracy of 91.77%. It is well-suited for managing intricate sequential patterns in supervised tasks because of its bidirectional nature, which allows it to learn from both past and future context. The model’s constant accuracy across classes is demonstrated by performance measures including precision, recall, and F1-score, which range from 0.87 to 1.0 in Fig 14 (a). and support the model’s efficacy in real-world classification settings. Its robustness is confirmed by the confusion matrix in Fig 14 (b), which offers a more detailed perspective and insights into certain prediction strengths and sporadic misclassifications. All things considered, BiLSTM is a very strong model for practical classification tasks, particularly those requiring reliable predictions and intricate pattern recognition. Its effectiveness in this situation highlights how beneficial supervised DL methods are at drawing insightful conclusions from difficult datasets. The BiLSTM model’s normalized confusion matrix is displayed in Fig 14(c). Normalization, which shows the percentage of accurate and inaccurate predictions for each class, makes classification results easier to understand than the raw confusion matrix. The off-diagonal values represent misclassification rates, whereas the diagonal values represent each class’s recall. For example, Class 0 achieves a recall of 0.99, Class 1 reaches 1.00, Class 2 records 1.00, Class 3 attains 0.99, and Class 4 achieves 0.98. In contrast, Class 5 shows a low recall of 0.26, with most of its misclassified samples (0.72) predicted as Class 0 and a small portion (0.02) as Class 3. Overall accuracy computed from the matrix consistent with the reported performance metrics. The accuracy and loss curves of the BiLSTM model for induction motor fault classification during training and validation are displayed in Fig 14 (d). The accuracy trend, which peaks at about 91.77%, is depicted in the left graph, where the validation accuracy continuously outperforms the training accuracy. The matching loss numbers are displayed in the right graph, both of which demonstrate a consistent decline with time. The training and validation curves’ tight alignment attests to efficient learning and sound generalization without overfitting.

#### 3.1.5. Stacked LSTM model.

**Fig 15:**
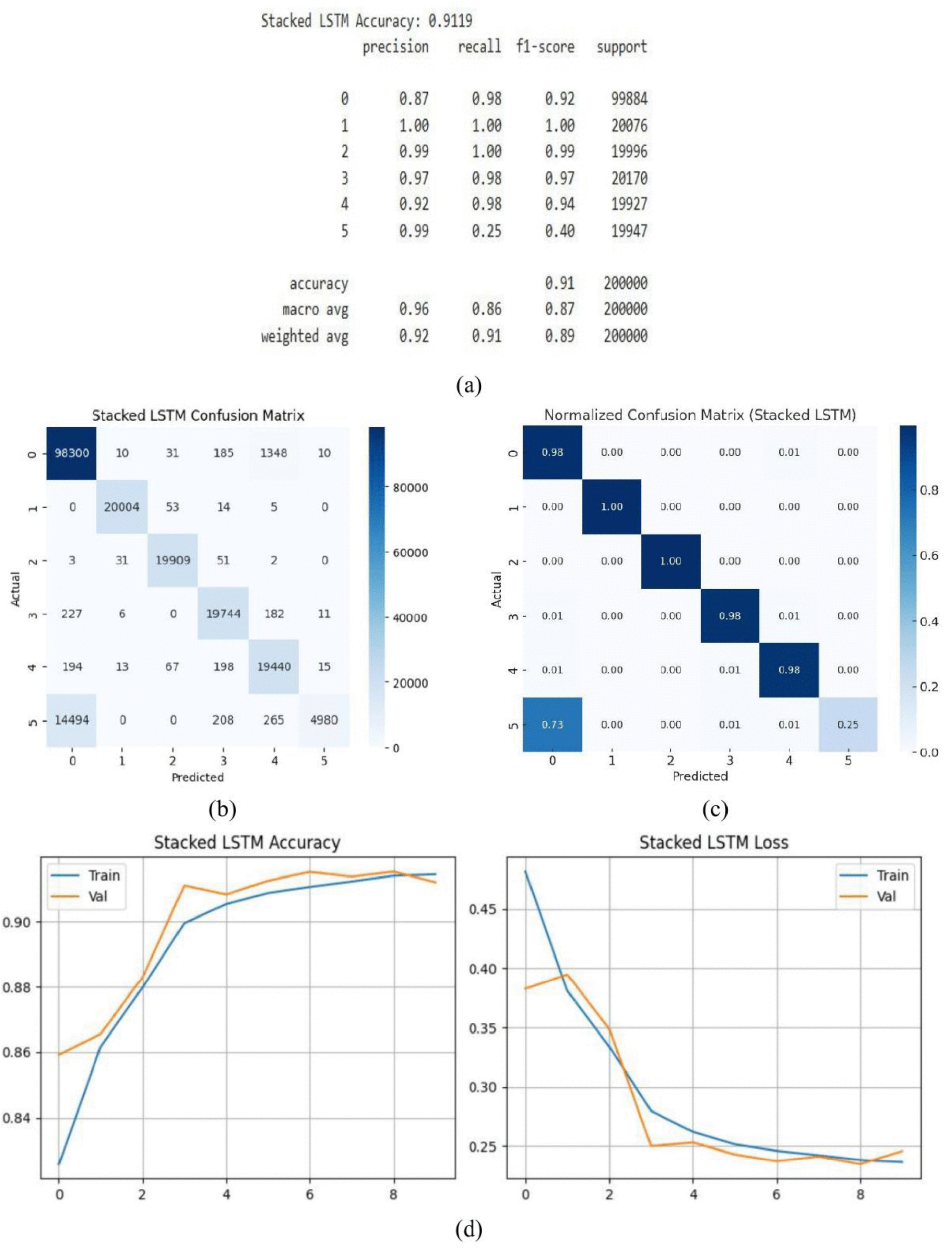
Stacked LSTM model (a) summary (b) confusion matrix (c) normalized confusion matrix (d)accuracy & loss curve.

With an accuracy of 91.19%, the Stacked LSTM model demonstrated its effectiveness in accurately classifying the great majority of cases. This impressive outcome demonstrates the model’s capacity to recognize and accurately depict intricate patterns in the dataset. The model can capture more complex temporal dependencies by gaining deeper representational power through the stacking of multiple LSTM layers. The accompanying assessment measures show consistent and dependable performance, with accuracy, recall, and F1-scores ranging from 0.87 to 1.0 across courses in Fig 15 (a). All things considered, the Stacked LSTM is a strong tool for supervised learning tasks containing intricate, time-based data. The confusion matrix in Fig 15 (b) provides a more thorough knowledge of the model’s efficacy by providing a thorough dissection of its predictions. Together with its steady near-perfect fits in a variety of scenarios, the Stacked LSTM model’s capacity to achieve such a high level of accuracy makes it an effective tool for complex pattern identification and accurate forecasting in practical applications. The normalized confusion matrix for the Stacked LSTM model is displayed in Fig 15(c). Normalization makes classification results easier to understand by showing the percentage of accurate and inaccurate predictions for each class, in contrast to the raw confusion matrix. Each class’s recall is represented by the diagonal numbers, while misclassification rates are shown by the off-diagonal values. For example, Class 0 achieves a recall of 0.98, Class 1 reaches 1.00, Class 2 records 1.00, Class 3 attains 0.98, and Class 4 achieves 0.98. In contrast, Class 5 shows a low recall of 0.25, with most of its misclassified samples (0.73) predicted as Class 0 and a small fraction (0.01) as Class 3 or Class 4. Overall accuracy computed from the matrix consistent with the reported performance metrics. The Stacked LSTM model’s training and validation accuracy and loss curves across ten epochs are shown in Fig 15 (d). The training accuracy grows consistently from about 82.7% to over 91.19%, as shown in the left subplot. During intermediate epochs, the validation accuracy also shows an increasing trend, marginally surpassing the training accuracy. This illustrates how well the model generalizes to new data. The matching loss curves are shown in the right subplot. As training goes on, there is a gradual convergence pattern after the training loss drops off dramatically in the first few epochs. The validation loss curve has a similar pattern, staying in tight alignment with the training loss. Effective learning and little overfitting are shown by the final loss stabilizing.

#### 3.1.6. GRU model.

**Fig 16:**
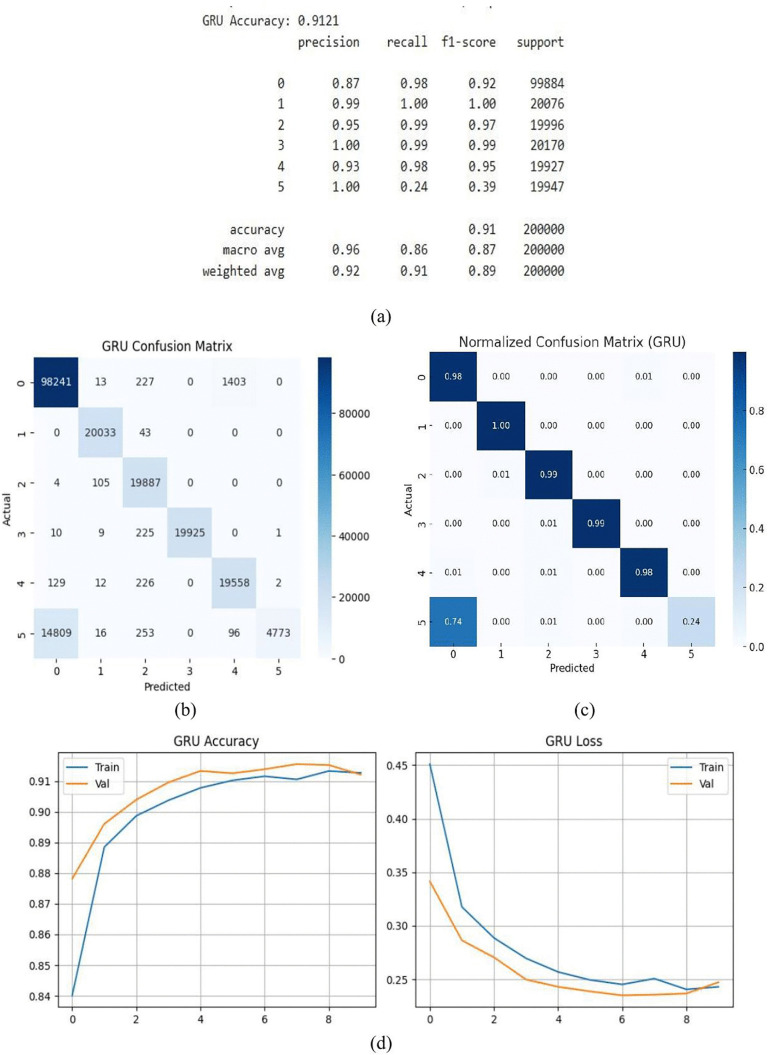
GRU model (a) summary (b) confusion matrix (c) normalized confusion matrix (d) accuracy & loss curve.

During testing, the GRU model classified most occurrences correctly, with an accuracy of 91.21%. This degree of accuracy demonstrates how well the model can learn and depict intricate temporal patterns in spite of its comparatively straightforward construction. Essential dependencies in sequential data are effectively captured by GRU because of its simplified gating mechanism. The assessment measures, precision, recall, and F1-scores, which range from 0.87 to 1.0 displays in Fig 16 (a) and show reliable, consistent predictions across courses, confirm this. GRU is a useful and dependable choice for supervised learning tasks using time-based data because of its capacity to manage intricate patterns while maintaining computational efficiency. The model’s efficacy is further clarified by the confusion matrix in Fig 16 (b), which provides a more in-depth analysis of the model’s predictions. Since the GRU model consistently produces near-perfect fits in a variety of scenarios and can attain such a high degree of accuracy, it becomes an invaluable tool for complex pattern identification. The GRU model’s normalized confusion matrix is displayed in Fig 16(c). Normalization, which shows the percentage of accurate and inaccurate predictions for each class, makes classification results easier to understand than the raw confusion matrix. The off-diagonal values represent misclassification rates, whereas the diagonal values represent each class’s recall. For instance, Class 0 records a recall of 0.98, Class 1 records a recall of 1.00, Class 2 records a recall of 0.99, Class 3 records a recall of 0.99, etc. The majority of Class 5’s misclassified samples (0.74) are expected to be Class 0, while a very tiny percentage (0.01) are anticipated to be Class 2 or Class 3. This results in a poor recall of 0.24. Total accuracy calculated from the matrix in accordance with the performance parameters that were supplied. The accuracy and loss behavior of the GRU model are shown in Fig 16 (d). The accuracy curves for training and validation both rise gradually, reaching a final accuracy of about 91.21%. The loss curves’ small gap indicates the GRU network’s effective training and strong generalization ability.

#### 3.1.7. CNN-GRU model.

**Fig 17:**
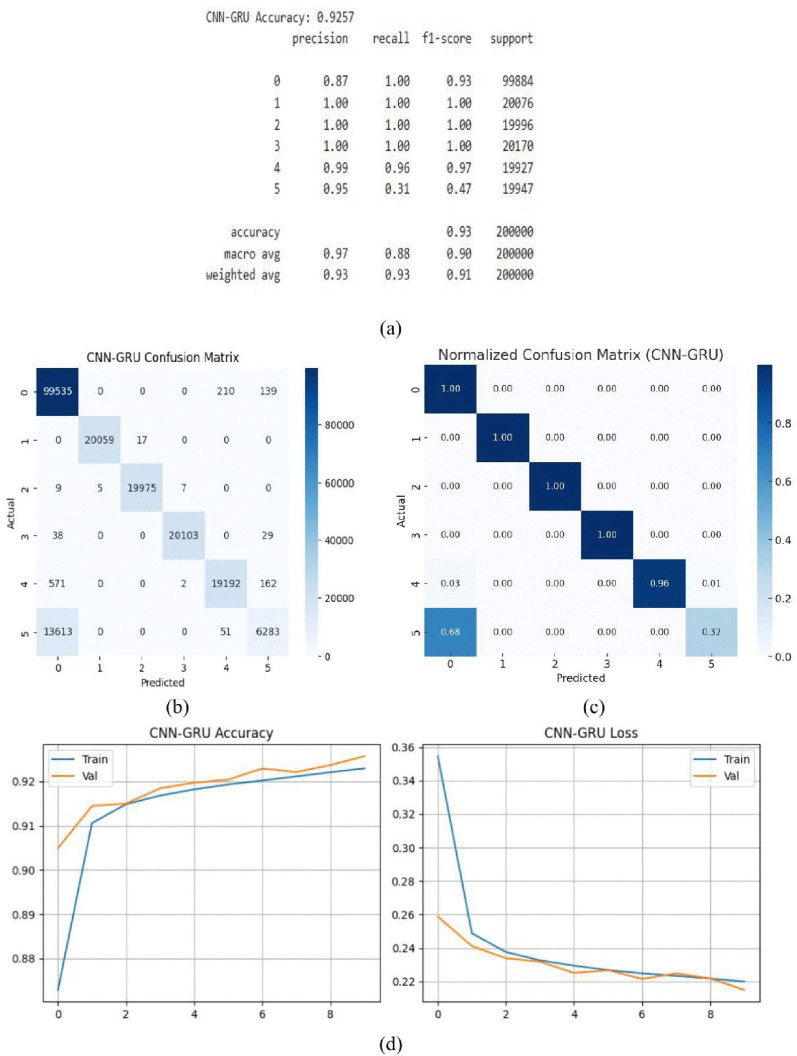
CNN-GRU model (a) summary (b) confusion matrix (c) normalized confusion matrix (d) accuracy & loss curve.

With a remarkable 92.57% accuracy rate, the CNN-GRU hybrid model accurately classified the vast majority of the samples. This illustrates how well the model combines the temporal learning capabilities of GRU with spatial feature extraction from CNN, enabling it to precisely handle intricate input patterns. Fig 17 (a) displays The model successfully learnt the complex structure of the dataset by utilizing GRU layers to capture sequential dependencies and convolutional layers to extract local features. Evaluation criteria that show constant and excellent performance across courses, including as accuracy, recall, and F1-scores ranging from 0.87 to 1.0, further confirm this. These outcomes demonstrate the model’s versatility and dependability for tasks requiring the recognition of both spatial and temporal patterns. The Fig 17 (b) displays our comprehension of the model’s efficacy is further improved by the confusion matrix, which provides a more thorough analysis of the predictions made by the model. When combined with its steady near-perfect fits in a variety of scenarios, the CNN-GRU model’s high accuracy level makes it an effective tool for complex pattern identification and accurate forecasting in practical applications. Fig 17(c) shows the normalized confusion matrix of the CNN-GRU model. Unlike the raw confusion matrix, normalization represents the proportion of correct and incorrect predictions for each class, making classification performance easier to interpret. The diagonal values correspond to the recall of each class, while the off-diagonal values indicate misclassification rates. For example, Class 0 achieves a recall of 1.00, Class 1 reaches 1.00, Class 2 records 1.00, Class 3 attains 1.00, and Class 4 achieves 0.96. In contrast, Class 5 shows a low recall of 0.32, with most of its misclassified samples (0.68) predicted as Class 0. Overall accuracy computed from the matrix consistent with the reported performance metrics. The CNN-GRU hybrid model’s performance is seen Fig 17 (d). Both the training and validation sets show a consistent increase in accuracy, which reaches about 92.57%. Training and validation loss are reduced smoothly and symmetrically in the loss plot, indicating stable and well-balanced training dynamics.

#### 3.1.8. CNN-LSTM model.

**Fig 18:**
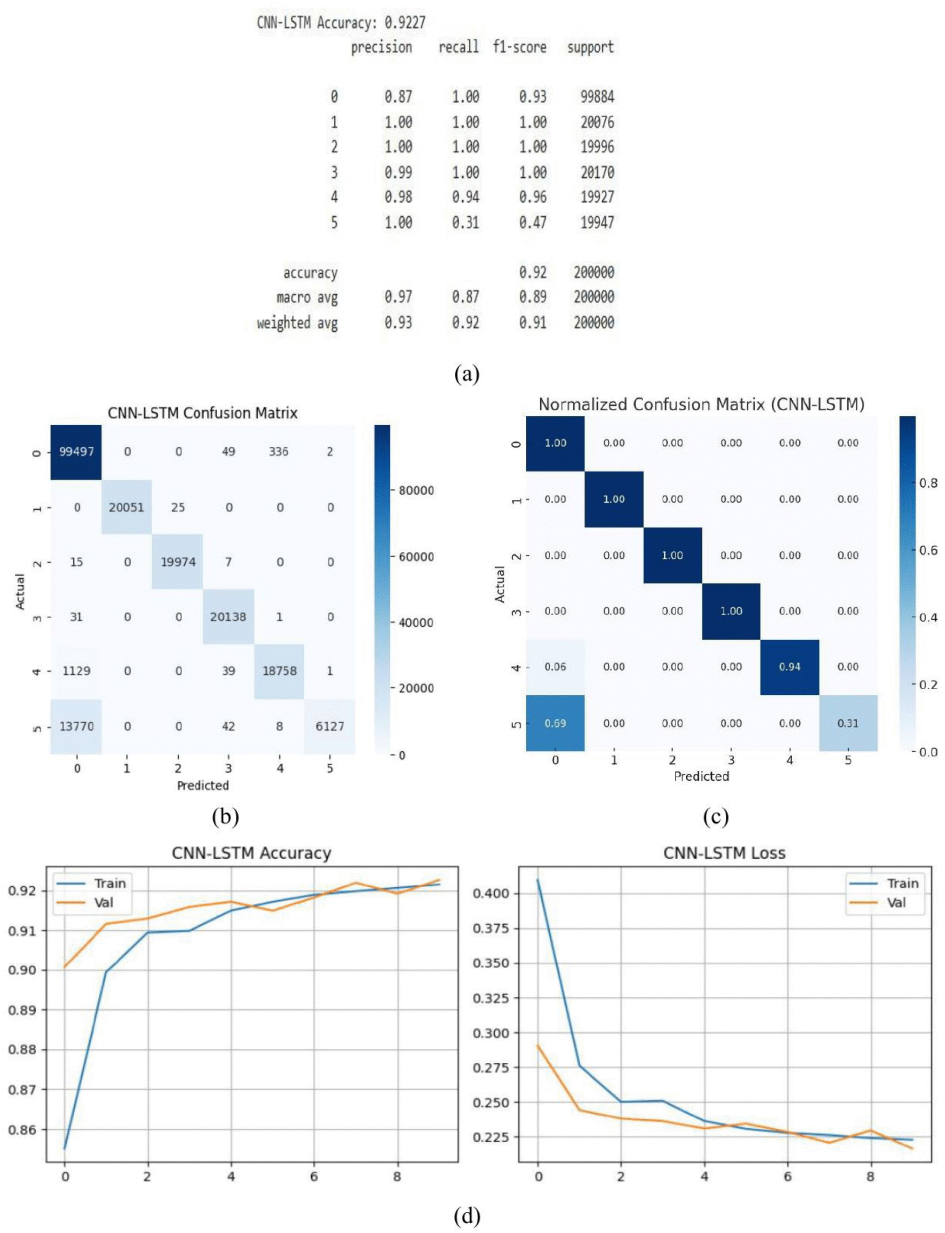
CNN-LSTM model (a) summary (b) confusion matrix (c) normalized confusion matrix (d) accuracy & loss curve.

The great majority of cases were successfully classified using the CNN-LSTM hybrid model, which had an accuracy of 92.27%. In order to efficiently learn both spatial and sequential patterns from the dataset, the model combines the advantages of LSTM layers for capturing temporal relationships with convolutional layers for feature extraction. Evaluation measures further confirm the model’s performance; as shown in Fig 18 (a), precision, recall, and F1-scores range from 0.87 to 1.0, demonstrating consistent accuracy across classes. These outcomes demonstrate how adaptable and dependable the model is when working with complicated data, which makes it ideal for applications requiring both temporal analysis and pattern identification. In order to properly assess the model’s overall performance, the confusion matrix in Fig 18 (b) offers a thorough breakdown of the prediction outcomes. In real-world applications, the CNN-LSTM model is a dependable and effective solution for sophisticated pattern recognition and precise forecasting because to its high accuracy and constant, nearly flawless categorization across numerous categories. The normalized confusion matrix of the CNN-LSTM model is displayed in Fig 18(c). Interpreting classification results is made simpler by normalization, which shows the percentage of accurate and inaccurate predictions for each class, in contrast to the raw confusion matrix. Misclassification rates are shown by the off-diagonal numbers, whilst the diagonal values represent each class’s recall. For example, Class 0 achieves a recall of 1.00, Class 1 reaches 1.00, Class 2 records 1.00, Class 3 attains 1.00, and Class 4 achieves 0.94. In contrast, Class 5 shows a low recall of 0.31, with most of its misclassified samples (0.69) predicted as Class 0. Overall accuracy computed from the matrix consistent with the reported performance metrics. The Fig 18 (d) displays the accuracy and loss curves for the hybrid CNN-LSTM model. With few variations, the training and validation accuracy approaches 92.27%. The model’s resilience in managing both spatial and sequential data is demonstrated by the uniform reduction of loss and the persistence of validation loss being marginally lower than training loss across epochs.

### 3.2. Performance comparison of DL models

For improved readability, normalized confusion matrices were also included in addition to the confusion matrices versions. These representations show the proportion of correct and incorrect classifications per class, thereby enabling easier comparison of classification performance across all fault categories and models. The Table 3. shows a comparison of different deep learning models in terms of accuracy, precision, recall, F1-score, inference time, latency, throughput, and memory usage, and the results clearly highlight the superior performance of hybrid CNN-based architectures. Among all models, the CNN-GRU emerged as the most effective, achieving the highest accuracy (92.57%), along with strong precision, recall, and F1 values (0.94, 0.93, 0.93). It also balanced computational efficiency with relatively low inference time (165.01s), reduced latency (105.56 ms), and high throughput (1212.05 samples/s), demonstrating its suitability for real-time environments and large-scale deployments. The CNN-LSTM followed closely with 92.27% accuracy, steady computational efficiency proving its ability to capture long-term temporal dependencies better than standalone recurrent networks, though requiring slightly higher inference time and memory. These results validate the effectiveness of combining CNN’s powerful feature extraction with the sequential learning capacity of GRU and LSTM, enabling the hybrids to outperform individual models. While the CNN-only model was computationally the fastest, with the lowest latency (101.98 ms) and the highest throughput (1254.50 samples/s), its accuracy (91.97%) remained marginally lower than the hybrids, making it a strong but less balanced alternative. Pure models such as LSTM, GRU, BiLSTM, and Stacked LSTM showed only moderate accuracy (91.19–91.77%) but suffered from higher latency and memory consumption, while the ANN was the weakest overall, with lower throughput and higher latency. Thus, the analysis confirms that CNN-GRU and CNN-LSTM provide the best overall trade-off, combining spatial and temporal learning to deliver robust, accurate, and efficient performance.

**Table 3:** Comparison of DL models.

Model	Accuracy (%)	Precision	Recall	F1 -score	Inference Time (s)	Latency (ms/batch)	Throughput (samples/s)	Memory (MB)
CNN-LSTM	92.27	0.94	0.93	0.93	167.64	107.24	1193.01	1996.25
CNN-GRU	92.57	0.94	0.93	0.93	165.01	105.56	1212.05	2020.71
CNN	91.97	0.93	0.92	0.92	159.43	101.98	1254.50	1895.50
BiLSTM	91.77	0.93	0.92	0.92	172.65	110.44	1158.42	2038.45
LSTM	91.36	0.92	0.91	0.91	169.34	108.33	1181.06	1953.12
GRU	91.21	0.92	0.91	0.91	166.67	106.62	1199.99	1972.21
Stacked LSTM	91.19	0.92	0.91	0.91	169.42	108.38	1180.50	2065.55
ANN	91.66	0.92	0.91	0.91	174.16	111.41	1148.39	1878.76

## 4. Conclusion

This study introduced a hybrid DL framework for real-time fault detection in SCIM, addressing the limitations of traditional diagnostic approaches. By integrating analytical motor modeling with advanced architectures, particularly CNN-GRU and CNN-LSTM, the proposed system effectively captured both spatial and temporal patterns in large-scale operational data. Using a balanced dataset of one million samples, the framework achieved strong results not only in classification accuracy (CNN-GRU reaching 92.57%) but also in precision, recall, and F1-score, while maintaining efficient computational metrics such as inference time, latency, and throughput. Among all tested architectures, CNN-GRU and CNN-LSTM provided the best trade-off between accuracy and efficiency. These results underscore the potential of hybrid DL for fault monitoring, enabling early detection of electrical, mechanical, and supply-related faults with minimal prior knowledge. The findings demonstrate that robust, scalable, and automated fault diagnosis is feasible for industrial applications, paving the way for self-aware and resilient manufacturing systems in modern smart industries. However, challenges remain in terms of computational demands, dataset annotation costs, and generalization across diverse motor types and operational scenarios.

Future work will prioritize optimizing model architectures for deployment in resource-constrained environments and expanding the dataset to include multi-modal sensor inputs. A critical direction is extending the framework to detect bearing faults, which represent one notable proportion of induction motor failures in real-world industries. Since bearing defects typically manifest in vibration and acoustic signals, incorporating such data modalities will enhance fault coverage. Additionally, validation with noisy industrial data, addressing sensor drift, load variation, and different fault severity levels, will further enhance robustness and ensure reliable applicability in real-world industrial settings. Direct benchmarking with state-of-the-art approaches remains an avenue for future, exploring transfer learning and lightweight hybrid models could make the solution more accessible to industries with limited infrastructure. In essence, this research marks a step toward intelligent, adaptive, and cost-effective motor health monitoring, promoting operational efficiency, reduced downtime, and sustainable industrial practices.

## Abbreviations

ANN: artificial neural network
BILSTM: bidirectional long short-term memory
CNN: convolutional neural networks
CWT: continuous wavelet transform
DL: deep learning
FEM: finite element method
GA: genetic algorithm
GBT: gradient boosted trees
GRU: gated recurrent unit
IMPSO: improved particle swarm optimization
K-NN: K-nearest neighbors
LSTM: long short-term memory
MCSA: motor current signature analysis
ML: machine learning
POUT: output power
RBFNN: radial basis function neural network
ReLU: rectified linear unit
RF: random forest
RMS: root mean square
RNN: recurrent neural networks
SCIM: squirrel-cage induction motor
TL: transfer learning
TPE: tree-structured Parzen estimator
VGG: visual geometry group.
